# Historical and Current Perspectives on the Systematics of the ‘Enigmatic’ Diatom Genus *Rhoicosphenia* (Bacillariophyta), with Single and Multi-Molecular Marker and Morphological Analyses and Discussion on the Monophyly of ‘Monoraphid’ Diatoms

**DOI:** 10.1371/journal.pone.0152797

**Published:** 2016-04-05

**Authors:** Evan W. Thomas, Joshua G. Stepanek, J. Patrick Kociolek

**Affiliations:** Department of Ecology and Evolutionary Biology and Museum of Natural History, University of Colorado at Boulder, Boulder, Colorado, 80309, United States of America; Institute of Botany, CHINA

## Abstract

This study seeks to determine the phylogenetic position of the diatom genus *Rhoicosphenia*. Currently, four hypotheses based on the morphology of the siliceous valve and its various ultrastructural components, sexual reproduction, and chloroplasts have been proposed. Two previous morphological studies have tentatively placed *Rhoicosphenia* near members of the Achnanthidiaceae and Gomphonemataceae, and no molecular studies have been completed. The position of *Rhoicosphenia* as sister to ‘monoraphid’ diatoms is problematic due to the apparent non-monophyly of that group, so hypotheses of ‘monoraphid’ monophyly are also tested. Using an analysis of morphological and cytological features, as well as sequences from three genes, SSU, LSU, and *rbc*L, recovered from several freshwater *Rhoicosphenia* populations that have similar morphology to *Rhoicosphenia abbreviata* (Agardh) Lange-Bertalot, we have analyzed the phylogenetic position of *Rhoicosphenia* in the context of raphid diatoms. Further, we have used topology testing to determine the statistical likelihoods of these relationships. The hypothesis that *Rhoicosphenia* is a member of the Achnanthidiaceae cannot be rejected, while the hypothesis that it is a member of the Gomphonemataceae can be rejected. In our analyses, members of the Achnanthidiaceae are basal to *Rhoicosphenia*, and *Rhoicosphenia* is basal to the Cymbellales, or a basal member of the Cymbellales, which includes the Gomphonemataceae. Hypothesis testing rejects the monophyly of ‘monoraphid’ diatoms.

## Introduction

Of the tremendous diversity found in the diatoms, one monophyletic group is the pennate diatoms [1]. Pennate diatoms may possess a raphe, a pair of slits through the glass cell wall that allows diatoms with this structure to micro-position themselves when in contact with a substratum. Some diatoms have a raphe system on both valves of their bipartite frustules (called biraphid diatoms), while others have a raphe system on one valve only (termed monoraphid diatoms). The systematic position of the raphid diatom genus *Rhoicosphenia* Grunow [2] has been the subject of considerable interest and debate from its inception as a distinct genus and for the subsequent 150 years. *Rhoicosphenia* was erected based on *Gomphonema curvata* Kützing [3] as the generitype and was differentiated from *Gomphonema* Ehrenberg [4] by having valves flexed about the transapical axis and shortened raphe branches on the convex valve. *Rhoicosphenia* was originally placed in the ‘monoraphid’ family Achnantheae [2], which also included *Achnanthes* Bory [5] *sensu lato*, (at the time both *Achnanthes sensu stricto* and *Achnanthidium* Kützing [6] were considered part of this genus) and *Cocconeis* Ehrenberg [7]. This systematic placement close to *Achnanthidium* within the ‘monoraphid’ diatoms has been followed by some workers [8–12].

After the description of *Rhoicosphenia*, Van Heurck [13] articulated what was the first alternate hypothesis regarding its phylogenetic position and placed it within the biraphid Tribe Gomphonemeae, citing similarities in chloroplast morphology between *Rhoicosphenia* and *Gomphonema*. Several diatomists of the 19^th^ and 20^th^ centuries agreed with this position [14, 15]. After Van Heurck, Mereschkowsky [16] noted that based on chloroplast structure, *Rhoicosphenia* was part of the raphid group Pyrenophoreae, which are united by a single chloroplast with a central pyrenoid. Within the Pyrenophoreae, Mereschkowsky also suggested the closest relative of *Rhoicosphenia* to be *Gomphonema* [16], with both genera being in the tribe Gomphonemeae. Mereschkowsky’s Pyrenophoreae was part of the larger group, the Monoplacatae, along with another group of note, the Heteroideae [16]. Genera included in the Pyrenophoreae and considered in our paper were *Anomoeoneis* Pfitzer [17], *Cymbella* Agardh [18], *Encyonema* Kützing [3], and *Placoneis* Mereschkowsky [19], while the Heteroideae included the genera *Cocconeis* and *Microneis* Cleve [20] (now *Achnanthidium*). Cleve [20] provided a less concrete placement of *Rhoicosphenia* due to his interpretation of ‘monoraphid’ diatoms as not a ‘natural’ group, i.e. polyphyletic, while Schütt [21] hypothesized it to be a ‘Bindeglied zwischen’ (translated as ‘link between’) *Gomphonema* and *Achnanthes*, and Schütt’s view was illustrated in Peragallo [8].

*Rhoicosphenia* and *Gomphonema*, are currently placed in the Cymbellales Mann [22], while Achnanthidium is placed in the Achnanthales Silva [23]. Round *et al*. [22] proposed the following genera to be in the Cymbellales: *Anomoeoneis* (Anomoeoneidaceae), *Placoneis*, *Cymbella*, *Encyonema* (Cymbellaceae), *Gomphonema*, *Didymosphenia* M. Schmidt in [24], *Gomphoneis* Cleve [25], and *Reimeria* Kociolek & Stoermer [26] (Gomphonemataceae), and *Rhoicosphenia* (Rhoicospheniaceae Chen & Zhu [12]). *Cymbopleura* Krammer [27], *Geissleria* Lange-Bertalot & Metzeltin [28], and *Encyonopsis* Krammer [29] were erected and remained in the Cymbellales and molecular analyses have supported their placement [30, 31], while several other genera are included in the order [22], but have not been formally analyzed with either morphological or molecular data. ‘Gomphonemoid’ diatoms include four genera in Kützing’s [6] Gomphonemataceae, but morphological and molecular analyses revealed that *Gomphonema* and *Gomphoneis* should be in the family, while *Didymosphenia* and *Reimeria* are more closely related to members of the Cymbellaceae [26, 31, 32]. Thus, for this paper, we consider only *Gomphonema* and *Gomphoneis* to be ‘gomphonemoid’ diatoms. When we refer to the Cymbellales we are doing so in the expanded sense of Round *et al*. [22], with inclusion of *Cymbopleura*, *Geissleria* and *Encyonopsis*, but excluding *Rhoicosphenia*, as we are testing its phylogenetic position.

Genera in the Achnanthales per Round *et al*. [22] include *Achnanthes* (Achnanthaceae), *Cocconeis* (Cocconeidaceae), and *Achnanthidium* (Achnanthidiaceae). These are often referred to as ‘monoraphid’ diatoms, due to the presence of a raphe system on one valve only, and over the past two decades several genera including *Karayevia* Round & Bukhtiyarova ex [33], *Lemnicola* Round & Basson [34], *Planothidium* Round & Bukhtiyarova [35], *Platessa* Lange-Bertalot in [36], *Psammothidium* Bukhtiyarova & Round [37], and *Rossithidium* Round & Bukhtiyarova ex [33] have been proposed and include many species assigned previously to *Achnanthidium* and other genera in this group. Molecular data have been generated for some of these taxa, and the position of *Achnanthes sensu stricto* has been shown [38–40] distinct from other ‘monoraphid’ genera, such as *Achnanthidium*, *Cocconeis*, and *Lemnicola*. Based on the distant phylogenetic position of *Achnanthes sensu stricto*, we will here take a narrower view of ‘monoraphid’ diatoms and include the genera *Achnanthidium*, *Cocconeis*, *Lemnicola*, *Planothidium*, and *Psammothidium*, but exclude *Achnanthes*. The distant phylogenetic position of *Achnanthes* relative to the other aforementioned monoraphid genera was proposed by Mereschkowsky [16] and has been supported by molecular phylogenies [41, 42]. Mereschkowsky [16] placed *Achnanthidium* (then *Microneis*) and *Cocconeis* into the Heteroideae, which excluded *Achnanthes*, so we will test whether *Rhoicosphenia* is part of a monophyletic group with taxa in the Heteroideae.

**Fig 1:**
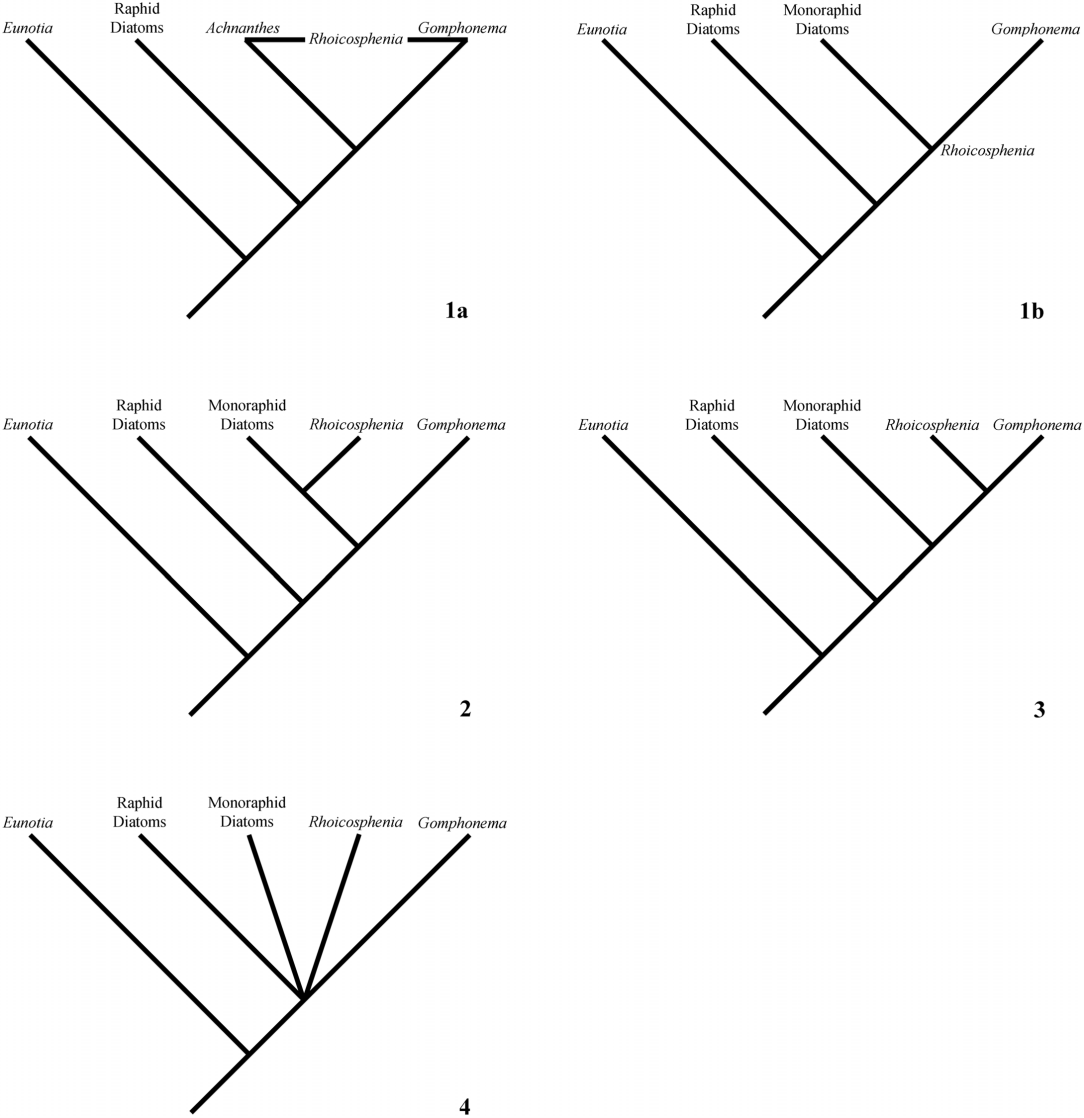
Summary of historical hypotheses.

In the 1980’s, there was substantial interest in the phylogenetic position of *Rhoicosphenia* [43– 48]. Mann [43] asserted four hypotheses for the systematic position of *Rhoicosphenia*, which are paraphrased as follows (Fig 1);

1.
  - *Rhoicosphenia* is an intermediate form between *Achnanthes* and *Gomphonema*, or,
  - The common ancestor of ‘monoraphid’ and ‘gomphonemoid’ genera,
2. *Rhoicosphenia* is a ‘monoraphid’ diatom,
3. *Rhoicosphenia* is related to *Gomphonema*, and
4. *Rhoicosphenia* is unrelated to ‘monoraphid’ and gomphonemoid diatoms.

Hypothesis 1 has two parts; (a) *Rhoicosphenia* is an intermediate form between *Achnanthes* and *Gomphonema*, and (b) is the common ancestor of both ‘monoraphid’ and gomphonemoid groups. Hypothesis 1a was proposed by Schütt [21] with *Rhoicosphenia* being the link between *Gomphonema* and *Achnanthes*, but we are unable to test the topology with our statistical methods and will therefore not statistically address the hypothesis in this paper. Hypothesis 1b is not testable with hypothesis testing techniques, since *Rhoicosphenia* would not occupy a position as a terminal taxon, but rather be placed at a node of divergence between ‘monoraphid’ and gomphonemoid diatoms. However, the hypothesis will be tested broadly in the context of the position of *Rhoicosphenia* compared to other genera. Hypothesis 2 [43] follows Grunow and Hustedt, with *Rhoicosphenia* being more closely related to ‘monoraphid’ diatoms. Hypothesis 3 [43] follows Van Heurck and Mereschkowsky and states that *Rhoicosphenia* is sister to *Gomphonema*. Finally, hypothesis 4 [43] most closely resembles Cleve’s hypothesis that the phylogenetic affinity of *Rhoicosphenia* to ‘monoraphid’ diatoms is due to polyphyletic origins of the ‘monoraphid’ condition, but also does not lend itself to hypothesis testing because we cannot place *Rhoicosphenia* in an unknown position in the tree.

In studying the morphology of *Rhoicosphenia* valves in detail, some of Mann’s [43] conclusions were that the valve symmetry of *Rhoicosphenia* is similar to *Gomphonema* and *Cymbella*, *Rhoicosphenia* valves are not similar to *Achnanthes* or *Cocconeis*, the chloroplasts of *Rhoicosphenia* are more similar to *Achnanthidium* than *Achnanthes* (and cites [16] chloroplast work), and *Rhoicosphenia* is unlike *Gomphonema* due to areolar occlusions differences [43]. Subsequently, Mann notes differences in sexual reproduction between the isogamous *Rhoicosphenia* and the physiological anisogamy of *Gomphonema* and *Cymbella* [44]. The conclusions of Mann’s final paper support the 4^th^ hypothesis, that *Rhoicosphenia* ‘clearly’ is not allied with ‘monoraphid’ diatoms, but belongs in an ‘isolated position’ near the gompho-cymbelloid diatoms within the Naviculales and offers an emended description of the family Rhoicospheniaceae [45].

Soon after Mann’s papers, a cladistic analysis of *Cocconeis*, *Mastogloia* Thwaites in [49], *Achnanthes sensu lato*, *Gomphonema*, and *Rhoicosphenia* was produced [48]. Using eleven morphological characters to test historical hypotheses similar to those in Mann [43], the analysis showed that *Rhoicosphenia* is more closely related to *Gomphonema*, with *Achnanthes sensu lato* as sister and *Cocconeis* more distantly related [48]. In that analysis, *Rhoicosphenia* did not occupy an undetermined position, but was sister to *Gomphonema* and only closely allied with one of the other ‘monoraphid’ genera, *Achnanthes sensu lato*. A more recent cladistic analysis using morphology that included *Rhoicosphenia* employed more characters (n = 35) and taxa (n = 49). This analysis placed *Rhoicosphenia* in an unresolved polytomy of raphid diatoms [50]. These subsequent results do not support Grunow’s hypothesis of relationship, based on his decision to place his ‘newly’ erected genus in the Achnantheae, and also rejects the hypothesis that *Rhoicosphenia* is sister to *Gomphonema*. The results showed that some members of Cymbellales *sensu* Mann in [22], (*Cymbella*, *Encyonema*, *Gomphonema*, and *Reimeria*) are a natural group, but *Anomoeoneis*, *Placoneis* and *Rhoicosphenia* were not allied with that group [50]. Also, the ‘monoraphid’ diatoms in that study, *Achnanthidium* and *Cocconeis*, formed a natural group, but *Rhoicosphenia* was excluded from that clade [50]. In terms of the four hypotheses forwarded by Mann, the study by [50] supports hypothesis 4, that *Rhoicosphenia* occupies an ‘enigmatic’ position in the raphid diatom phylogeny [43, 45]. Cox [51] discussed several morphological characters of *Achnanthes sensu stricto* and suggested it belongs in the Mastogloiales Mann in [22], rather than Achnanthales, again casting doubt on the monophyly of ‘monoraphid’ diatoms, supporting proposals made at the turn of the 20^th^ century [16, 20]. *Rhoicosphenia* is also interesting because two of its potential phylogenetic positions, ‘monoraphid’ or *Gomphonema* (Cymbellales), are consistently returned as sister taxa in molecular analyses [1, 38–40, 42, 52–54], but many of these analyses are focused on other questions and have not discussed this relationship [22, 31, 55–58].

Two additional hypotheses are added that are not strictly related to *Rhoicosphenia*, but more broadly to ‘monoraphid’ diatoms. The first, H_5_, addresses the issue of whether or not all ‘monoraphid’ diatoms are monophyletic. Several molecular and one morphological [50] have suggested that this is not the case, as *Achnanthes sensu stricto* is not part of a monophyletic group with the other ‘monoraphid’ diatoms, such as *Achnanthidium* and *Cocconeis*, and in fact is quite distantly related to them. The second, H_6_, tests the hypothesis, forwarded by Cox [51], that *Achnanthes sensu stricto* is closely related to the genus *Mastogloia*.

The major goal of this project is to use single and multi-marker molecular analyses, as well as analysis of morphological data to determine the systematic position of *Rhoicosphenia* in the diatom tree of life within the context of previous taxonomic hypotheses.

## Materials and Methods

### Molecular Analyses

#### Taxon collections

Three *Rhoicosphenia* populations were isolated from freshwater streams into monoculture via micro-pipette serial dilution from collections made in California, Colorado and Oregon, USA, and were grown in freshwater WC medium [59]. After isolation, the cultures were maintained at a temperature of approximately 25C, with a 12:12 light dark cycle at an irradiance of 50 μmol cm^-2^ s^-1^. The other 4 sets of sequences were obtained via a Chelex extraction from colonies found in live samples. Colonies were chosen to ensure that DNA was obtain from one genetic clonal line. Table 1 contains information on sampling locations of sequenced specimens. Samples in California were collected with a Scientific Collecting Permit from the California Department of Fish and Wildlife, issued to Evan W. Thomas. The Oregon Department of Fish and Wildlife and Colorado Department of Natural Resources did not require permits for microalgal collections. All collections were made from state, county, and city parks, or from waterways accessible from public roads and no field sites had endangered or protected species. Prepared diatom slides, referenced by Collection number in Table 1, containing sequenced population are housed in the Kociolek Diatom Collection, University of Colorado, Museum of Natural History, Boulder, Colorado, USA.

**Table 1:** Sampling location information *Rhoicosphenia* populations sequenced including species, ID, State, County, Site Name, Latitude, Longitude, Type, and Collection number.

Taxon Name	ID	State	County	Site Name	Latitude	Longitude	Type	Collection number
*Rhoicosphenia* cf. *abbreviata* (Agardh) Lange-Bertalot	1 EWT	CO	Boulder	Golden Ponds	40.1674	-105.1417	Culture	10927
*Rhoicosphenia* cf. *abbreviata* (Agardh) Lange-Bertalot	2 EWT	CO	Boulder	Gaynor Lake	40.1168	-105.1056	Culture	10926
*Rhoicosphenia stoermeri* E.W. Thomas & Kociolek	3 EWT	CA	Santa Barbara	Mission Creek	34.4126	-119.6913	Chelex	9507
*Rhoicosphenia* cf. *abbreviata* (Agardh) Lange-Bertalot	4 EWT	CA	San Diego	Penasquitos Creek	32.9439	-117.08	Chelex	9533
*Rhoicosphenia* cf. *abbreviata* (Agardh) Lange-Bertalot	37 EWT	OR	Hood River	Hood River	45.7101	-121.5071	Chelex	9798
*Rhoicosphenia* cf. *abbreviata* (Agardh) Lange-Bertalot	80 EWT	OR	Linn	Willamette River	44.6380	-123.1602	Chelex	9829
*Rhoicosphenia* cf. *abbreviata* (Agardh) Lange-Bertalot	94 EWT	OR	Lane	McKenzie River	44.0558	-122.8281	Culture	9816

Seven *Rhoicosphenia* populations were sequenced for this analysis with 7 isolates yielding partial 18S small subunit rDNA (SSU) sequences, 6 sequences from the D1–D2 region of the 28S large subunit rDNA (LSU), and 4 sequences from the chloroplast encoded large subunit of RUBISCO (*rbc*L). Only three populations yielded sequences for all 3 markers. The list of populations studied, including taxon name, ID, and GenBank accession numbers is presented in Table 2. Additionally, GenBank was used to obtain an additional 140 sequences for SSU, 80 sequences for LSU, and 100 sequences for *rbc*L and a list of these taxa are included as supplemental document S1 Table. The concatenated three marker tree includes 3 *Rhoicosphenia* sequences and 78 GenBank sequences (S2 Table).

**Table 2:** *Rhoicosphenia* populations sequenced including name, ID, molecular marker sequences available, and GenBank accession numbers.

Name	ID	SSU	LSU	*rbc*L
*Rhoicosphenia* cf. *abbreviata*	1 EWT	KU965564	KU965571	KU965577
*Rhoicosphenia* cf. *abbreviata*	2 EWT	KU965565	KU965572	KU965578
*Rhoicosphenia stoermeri*	3 EWT	KU965566	KU965573	KU965579
*Rhoicosphenia* cf. *abbreviata*	4 EWT	KU965567	KU965574	n/a
*Rhoicosphenia* cf. *abbreviata*	37 EWT	KU965568	KU965575	n/a
*Rhoicosphenia* cf. *abbreviata*	80 EWT	KU965569	n/a	KU965580
*Rhoicosphenia* cf. *abbreviata*	94 EWT	KU965570	KU965576	n/a

#### DNA extraction amplification and sequencing

A Chelex 100^®^ method [60] was used to extract DNA from monocultures and was modified to a volume of 20 μL Chelex for colonies of *Rhoicosphenia*. The molecular markers chosen, include the conserved (SSU) and variable (LSU, *rbc*L), which have been shown to provide order [1, 38, 42, 55] and species [61–63] level resolution. Further, due to the widespread use of these markers in diatom phylogenetics [1, 31, 38–40, 42, 53, 55, 61, 63], it allowed for the broadest taxon sampling of non-*Rhoicosphenia* GenBank sequences from the raphid diatoms. Primers used in amplification and sequencing of these markers are listed in Table 3.

**Table 3:** Primers used in amplification and sequencing of SSU, LSU, and *rbc*L. ^a^ Forward PCR amplification primer, ^b^ Reverse PCR amplification primer.

Primer Name	Primer Sequence (5′ to 3′)	Reference
SSU Primers		
SSU1^a^	AAC CTG GTT GAT CCT GCC AGT	[64]
SSU850+	GGG ACA GTT GGG GGT ATT CGT A	[38]
SSU870-	TAC GAA TAC CCC CAA CTG TCC C	[38]
ITS1DR^a^	CCT TGT TAC GAC TTC ACC TTC C	[65]
LSU Primers		
D1R^a^	ACC CGC TGA ATT TAA GCA TA	[66]
D2C^b^	CCT TGG TCC GTG TTT CAA GA	[66]
*rbc*L Primers		
*rbc*L66+^a^	TTA AGG AGA AAT AAA TGT CTC AAT CTG	[61]
*rbc*L404+	GCT TTA CGT TTA GAA GAT ATG	[38]
*rbc*L1255-	TTG GTG CAT TTG ACC ACA GT	[61]
dp7-^a^	AAA SHD CCT TGT GTW AGT YTC	[67]

Using GE Healthcare illustra Ready-To-Go^™^ PCR beads (GE Healthcare Biosciences, Pittsburgh, Pennsylvania) following the manufacturer’s instructions, all markers were amplified by polymerase chain reaction (PCR). PCR was performed in an Eppendorf Mastercycler^®^ using the program: 94 C for 3:30, 36 cycles of 94 C for 50 seconds, 52 C for 50 seconds, 72 C for 80 seconds, with a final extension at 72 C for 15 minutes. After amplification, the PCR products were purified with ExoSAP-IT (Affymetrix, Santa Clara, California) using the manufacturers protocol. Purified PCR products were sequenced at Functional Biosciences, Inc. (Madison, Wisconsin) and Geneious ver. 5.6 [68] was used to assemble and edit sequences. Sequences for the seven *Rhoicosphenia* taxa included in this analysis are deposited in GenBank and accession numbers for SSU, LSU, and *rbc*L sequences are listed in Table 1.

#### Sequence alignment and phylogenetic analysis

A muscle alignment algorithm [69] in Geneious was used for all alignments. The three molecular markers were aligned separately prior to concatenation in the two and three-molecular marker alignments. The ends were trimmed from each of the alignments to minimize missing characters. A variable 63 base pair region of SSU, corresponding to region 579–641 in the initial alignment, was removed due to the ambiguity in the alignment, creating a final trimmed length of 1566 sites. The final trimmed length of LSU was 604 base pairs and *rbc*L had a final trimmed length of 799 base pairs. The three-marker concatenated alignment for 81 taxa was 2969 sites. The SSU alignment included 140 non-*Rhoicosphenia* taxa with representatives from all available raphid diatom orders *sensu* [22]. The LSU and *rbc*L alignments included less taxa, but attempted to maintain coverage of raphid diatom groups based on available sequences. The number of taxa included in alignments are as follows: SSU—147; LSU—86; *rbc*L—104; SSU + LSU—85; SSU + *rbc*L—97; LSU + *rbc*L—81; and SSU + LSU + *rbc*L—81. To understand the position of *Rhoicosphenia* in the diatom tree of life, both maximum likelihood (ML) and Bayesian analyses were performed all single, two-gene, and three-molecular marker alignments. The alignments are provided as supplemental files (S1 File: SSU + LSU + *rbc*L; S2 File: SSU + LSU; S3 File: SSU + *rbc*L; S4 File: LSU + *rbc*L; S5 File: SSU; S6 File: LSU; S7 File: *rbc*L) and have also been uploaded to figshare (https://figshare.com) and their DOI is 10.6084/m9.figshare.3115522. All seven alignments were analyzed using the general time reversible (GTR) model with a gamma distribution (Γ) and a proportion of invariable sites (I) [1, 40]. SeaView version 4.3.4 [70] was used to perform maximum likelihood (ML) analysis with PhyML version 3.0 [71] using the GTR+ Γ+I model with four rates classes and 500 bootstrap replicates to estimated branch support. MrBayes version 3.2.1 [72] was used to perform Bayesian analyses. Analyses were run using the default settings and a GTR+Γ+I model with four rate classes. The single and two-molecular marker alignments were run for 10 million generations with a burn-in of 2 million generations, and the three-molecular marker alignment was run for 30 million generations with a burn-in of 6 million generations; all alignments were analyzed using two runs of four MCMC chains sampled every 1000 generations. Maximum likelihood phylograms are presented in this paper and nodes are labelled with maximum likelihood bootstrap values (BS)/Bayesian posterior probabilities (BPP) reported as percentages. In situations where the ML and Bayesian trees are incongruent, the Bayesian node support is denoted as (-).

#### Hypothesis testing

Hypotheses concerning the monophyly of *Rhoicosphenia* were tested using tree likelihoods and the Approximately Unbiased (AU) test [73]. For the test using the two and three-molecular marker alignments, an unconstrained tree (H_0_) was tested against four constrained alternative topologies:

H_2a_: *Rhoicosphenia* is in a monophyletic clade with all members of the Heteroideae, *sensu* [2, 10, 16],
H_2b_: *Rhoicosphenia* is monophyletic with the clade of Heteroideae that contains *Achnanthidium*,
H_2c_: *Rhoicosphenia* is monophyletic with the clade of Heteroideae that does not contain *Achnanthidium*, and
H_3_: *Rhoicosphenia* and *Gomphonema* form a monophyletic group, *sensu* [13, 16].

For the tests using single molecular marker trees, the unconstrained tree (H_0_) was tested against five constrained alternative topologies:

H_2a_: *Rhoicosphenia* is in a monophyletic clade with all members of the Heteroideae diatoms,
H_2b_: *Rhoicosphenia* is monophyletic with the clade of Heteroideae that contains *Achnanthidium*,
H_2c_: *Rhoicosphenia* is monophyletic with the clade of Heteroideae that does not contain *Achnanthidium*,
H_3a_: *Rhoicosphenia* and *Gomphonema* ‘clade 1’ (*Gomphonema* and *Gomphoneis*) form a monophyletic group, and
H_3b_: *Rhoicosphenia* and *Gomphonema* ‘clade 2’ (*G*. *micropus*) form a monophyletic group. Hypotheses 1 and 4 were unable to be testing using this method.

Finally, for the SSU, *rbc*L, and SSU + *rbc*L alignments, we also are testing:

H_5_: Are all ‘monoraphid’ diatoms monophyletic? The genera included in this test are *Achnanthes*, *Achnanthidium*, *Cocconeis*, *Lemnicola*, *Planothidium*, and *Psammothidium*. Some of the molecular marker combinations have different taxa, but are limited to these genera. And,
H_6_: Are the genera *Achnanthes* and *Mastogloia* monophyletic?

RAxML ver. 8.0.26 [74] and the graphical user interface raxmlGUI ver. 1.3.1 [75] were used to generate maximum likelihood trees from the unconstrained and constrained alignments for hypotheses 2 and 3 (A & B), using GTR+ Γ+I model. The probability that the alternative topologies were as likely as the null topology (unconstrained tree) was tested by calculating per site log likelihood values using RAxML and implementing the AU in the program CONSEL using default settings [76]. In CONSEL the AU test compares a hypothesized tree topology to a set of trees generated through a multi-scale bootstrap technique of per site log likelihoods. A statistically significant result, p-value less than or equal to 0.05, means that the hypothesized tree topology can be rejected, while a p-value greater than 0.05 does not allow the rejection of the hypothesized constrained tree.

### Morphological analyses

The taxa, character matrix, and character states used in this analysis were published in [50]. Our analysis used 33 of the 49 taxa published in [50] to maximize taxa shared between our morphological and molecular analyses. The characters used, as well as their coding, has been left unchanged from the original dataset [50], but we ran all data, protoplast and frustule, together in our analysis. The explanation and coding of characters can be found in S3 Table and the taxon and character matrix is presented in S4 Table. Phylogenetic analysis was performed in PAUP* 4.0b10 [77], and all 35 characters were unordered and equally weighted. Trees were generated using the branch-and-bound search option to determine the 200 most parsimonious trees that were then used to compute a strict consensus tree, which can be found as S1 Fig.

## Results

### Molecular Phylogenies

**Fig 2:**
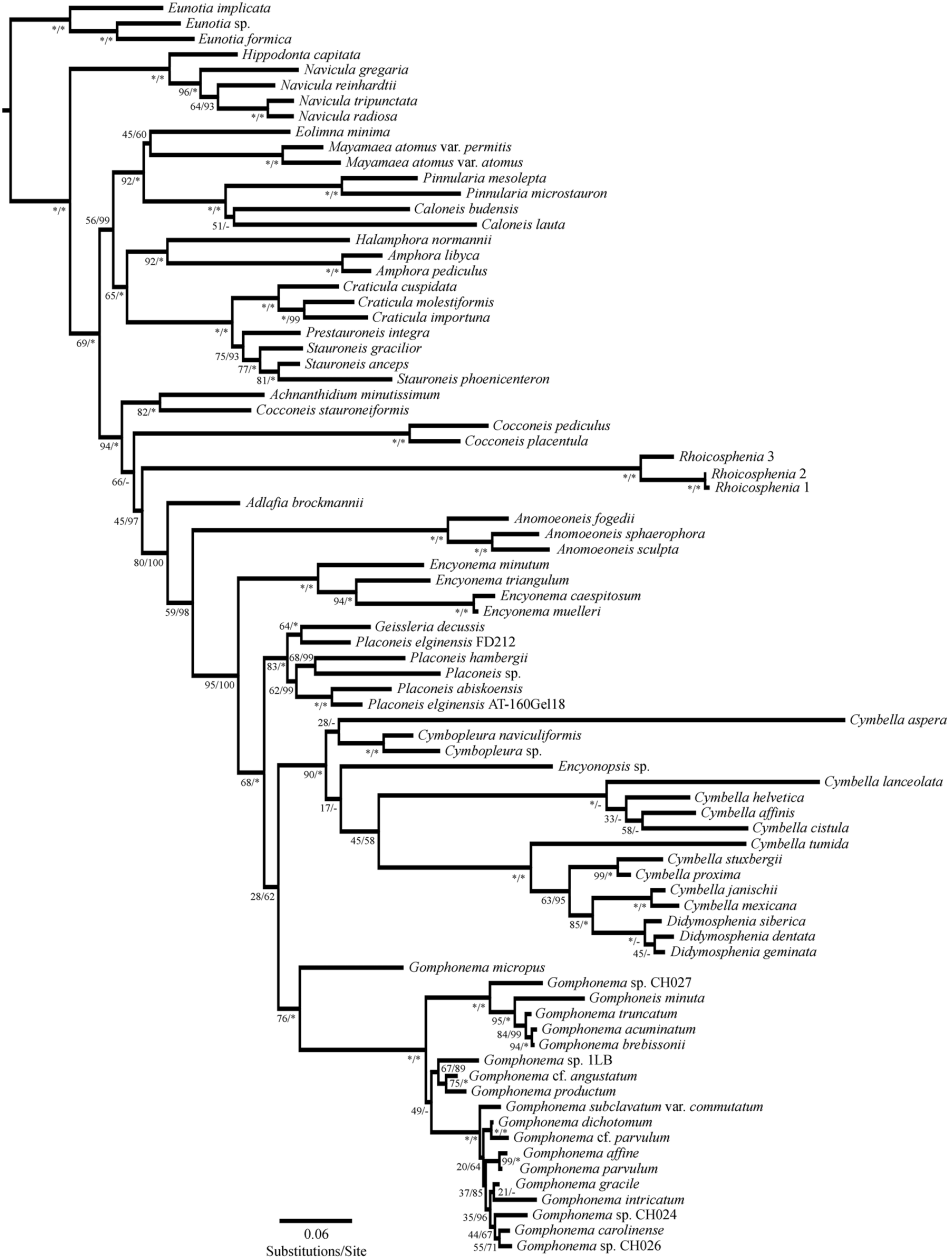
Maximum likelihood phylogram from three-marker concatenated alignment. Node support values are for maximum likelihood bootstrap values (500 bootstraps)/Bayesian posterior probability (as a percentage). “*” = 100, “-” = node incongruent between the two analyses.

In the analysis of the three-molecular marker concatenated alignment (Fig 2), both the ML and Bayesian analyses support a clade consisting of ‘monoraphid’ diatoms, members of the Cymbellales *sensu lato*, and *Rhoicosphenia*, to the exclusion of all other diatoms. In the ML three-molecular marker concatenated tree, *Rhoicosphenia* is not sister to *Cocconeis*, but is sister to the Cymbellales clade, with *Achnanthidium* and *Cocconeis* forming a grade basal to *Rhoicosphenia*. In the Bayesian three-molecular marker concatenated tree, *Achnanthidium* and *Rhoicosphenia* + *Cocconeis* are a ‘monoraphid’ grade basal to the Cymbellales.

Supplemental phylogenies (S2a and S2b Fig, S3a and S3b Fig, S4a and S4b Fig, S5a and S5b Fig, S6a and S6b Fig, S7a and S7b Fig) have been uploaded to figshare (https://figshare.com), their DOI is 10.6084/m9.figshare.3115531, and they can be opened with appropriate tree viewing software, such as FigTree v1.3.1, with file S##a being the Maximum Likelihood tree, and S##b being the Bayesian tree.

When concatenated, the two nuclear markers, SSU and LSU, show consistent topologies in both ML and Bayesian analyses (S2 Fig). *Rhoicosphenia* strains are monophyletic, and sister to *Anomoeoneis*, that clade is sister to a large portion of the Cymbellales, including the genera *Encyonema*, *Cymbella*, *Cymbopleura*, *Didymosphenia*, *Geissleria*, *Placoneis*, *Gomphonema*, and *Gomphoneis*. Basal to the clade containing *Rhoicosphenia* and the aforementioned genera is *Adlafia* Moser, Lange-Bertalot & Metzeltin [78], and sister to *Adlafia* + *Rhoicosphenia* + Cymbellales is a basal grade of the ‘monoraphid’ genera *Achnanthidium* and *Cocconeis*.

ML and Bayesian analyses recover congruent topologies for SSU and *rbc*L when concatenated (S3 Fig). *Rhoicosphenia* strains are sister to *Cocconeis placentula* and *C*. *pediculus*, and the other ‘monoraphid’ taxa (*C*. *stauroneiformis*, *Lemnicola hungarica*, and *Achnanthidium minutissimum*) + *Rhoicosphenia* and the two *Cocconeis* are represented as a grade of taxa basal to the Cymbellales. These analyses show *Adlafia* as basal to the Cymbellales. The other ‘monoraphid’ taxa in these analyses, *Achnanthes sensu stricto* (four sequences), are not closely related to the previously mentioned ‘monoraphid’ diatoms and *Rhoicosphenia*.

LSU and *rbc*L results (S4 Fig) recover a monophyletic clade consisting of *Rhoicosphenia* + *Cocconeis placentula* and *C*. *pediculus* + *Achnanthidium minutissimum*, however, *C*. *stauroneiformis* is not part of that group. The clade of *Rhoicosphenia* + *C*. *placentula* and *C*. *pediculus* + *A*. *minutissimum* is not sister to the Cymbellales, however there is very low bootstrap support (44) for the node separating them from the intermediate clade made of biraphid naviculoid diatoms.

Both ML and Bayesian SSU analyses (S5 Fig) provide congruent results with the concatenated alignment that the genus *Rhoicosphenia* is basal to the Cymbellales. The SSU topology shows a well-supported (95 ML BS) lineage consisting of ‘monoraphid’ genera and the Cymbellales. *Cocconeis* and *Achnanthidium*, two ‘monoraphid’ genera, are non-monophyletic and are basal to a clade consisting of *Rhoicosphenia* + Cymbellales. The node where *Rhoicosphenia* splits from the Cymbellales has a bootstrap value of 45.

LSU results (S6 Fig) recover a topology where *Rhoicosphenia* is sister to two *Cocconeis* species, with another *Cocconeis* species sister to *Achnanthidium* and those two are not sister to *Rhoicosphenia* + *Cocconeis*. However *Rhoicosphenia* + *Cocconeis* are not sister to the Cymbellales, and are in a weakly supported (3 ML BS) clade with naviculoid diatoms. The Cymbellales clade recovered is similar to the clade in the three molecular marker and SSU analysis.

*rbc*L sequences result (S7 Fig) in a topology similar to the LSU analysis in that *Rhoicosphenia* is sister to *Cocconeis*. Unlike SSU, the *rbc*L phylogeny has more ‘monoraphid’ taxa (excluding *Achnanthes sensu stricto*) that form a weakly supported clade (10 ML BS) sister to the Cymbellales. Unlike LSU, *rbc*L does not result in a polytomy, but assigns branching order with *Rhoicosphenia* sister to *Cocconeis*, which together are sister to the Cymbellales.

### Hypothesis Testing on Molecular Phylogenies

Full results of hypothesis testing for all seven alignments; SSU, LSU, *rbc*L, SSU + LSU, SSU + *rbc*L, LSU + *rbc*L, and SSU + LSU + *rbc*L; can be found in Table 4. In testing alternate constrained topologies against the unconstrained phylogeny, examining the three molecular marker concatenated tree, we cannot reject H_2a_: that *Rhoicosphenia* is a Heteroideae diatom, H_2b_: that *Rhoicosphenia* is sister to *Achnanthidium*, and H_2c_: that *Rhoicosphenia* is sister to *Cocconeis*. The hypothesis that *Rhoicosphenia* is sister to *Gomphonema* (H_3_), could be rejected (p = 0.029).

**Table 4:** Summary of Hypothesis Testing Results. The first column states the molecular markers for the phylogeny being tested, while the first row represents the hypothesis being tested. The values in the table are the p-values from the Approximately Unbiased (AU) test [73], and hypotheses that can be rejected based on the AU test are indicated with a “*”.

	H_0_	H_2a_	H_2b_	H_2c_	H_3_	H_3a_	H_3b_	H_5_	H_6_
**SSU, LSU, *rbc*L**	0.424	0.310	0.109	0.790	0.023*				
**SSU, LSU**	0.629	0.307	0.331	0.609	0.042*				
**SSU, *rbc*L**	0.819	0.189	0.582	0.033*	0.231			6e-5*	0.125
**LSU, *rbc*L**	0.367	0.257	0.843	0.199	0.040*				
**SSU**	0.604	0.628	0.210	0.491		0.265	0.228	6e-48*	8e-6*
**LSU**	0.551	0.487	0.432	0.585		0.333	0.300		
***rbc*L**	0.650	0.481	0.612	0.019*		0.225	0.188	4e-5*	0.108

In the SSU + LSU analysis, we can only reject hypothesis 3, that *Rhoicosphenia* is sister to *Gomphonema* (p = 0.042).

For SSU + *rbc*L, we can reject H_2c_, that *Rhoicosphenia* is sister to *Cocconeis* (p = 0.033), and H_5_, that all ‘monoraphid’ diatoms are monophyletic (p < 0.001).

For LSU + *rbc*L, we can only reject hypothesis 3, that *Rhoicosphenia* is sister to *Gomphonema* (p = 0.040).

For SSU, we can reject H_5_, that all ‘monoraphid’ diatoms are monophyletic (p < 0.001), and also reject H_6_, that *Achnanthes sensu stricto* and *Mastogloia* are sister taxa (p < 0.001).

For LSU, we cannot reject any of the alternative hypotheses, H_2a,b,c_ or H_3a,b_.

For *rbc*L, we can reject H_2c_, that *Rhoicosphenia* is sister to *Cocconeis* (p = 0.019), and H_5_, that all ‘monoraphid’ diatoms are monophyletic (p < 0.001).

### Morphological Phylogeny

The strict consensus tree of the 200 trees returned from the branch-and-bound parsimony analysis was similar to the consensus tree using all data from [50]. Our tree (S7 Fig) returned *Rhoicosphenia* in an unresolved polytomy of 20 taxa, however within that polytomy members of the same genus did group together. Although our tree was unable to resolve relationships with any more detail than [50], we are still including the tree in this paper. The consistency (*CI*) and retention indices (*RI*) from our analysis, *CI* = 0.4727 & *RI* = 0.7434, are similar to those of [50], *CI* = 0.39 & *RI* = 0.77.

## Discussion

The results of the molecular analyses from this study provide insights into the evolution of the ‘monoraphid’ condition, and also lend support to the Cymbellales *sensu* Mann in [22], with both of these results having implications for the systematic position of *Rhoicosphenia*. First, SSU + *rbc*L (S3 Fig), SSU (S5 Fig), and *rbc*L (S7 Fig), do not support a monophyletic lineage of ‘monoraphid’ diatoms of the genera *Achnanthes*, *Achnanthidium*, *Cocconeis*, *Lemnicola*, *Planothidium*, and *Psammothidium* (Table 4). Past molecular results have indicated that *Achnanthes* is more closely related to the Bacillariales than the other genera previously listed [38–40, 42, 52–55], however Cox [51] hypothesized that *Achnanthes sensu stricto* and *Mastogloia* are sister taxa. Hypothesis testing for monophyly of these genera in the analyses of SSU + *rbc*L, SSU, and *rbc*L yields mixed results with SSU rejecting that relationship, while *rbc*L and SSU + *rbc*L failed to reject that relationship (Table 4). In light of these results, instead of testing the position of *Rhoicosphenia* against the non-monophyletic ‘monoraphid’ diatoms, we tested its position against the Heteroideae [16] consisting of the families Achnanthidiaceae (*Achnanthidium*, *Lemnicola*, *Planothidium*, and *Psammothidium*) and Cocconeidaceae (*Cocconeis*).

Our three-molecular marker analysis yields a well-supported relationship with *Rhoicosphenia* as sister to a monophyletic clade of the Cymbellales, and a grade of ‘monoraphid’ taxa including *Achnanthidium* and *Cocconeis* is sister to *Rhoicosphenia* + the Cymbellales (Fig 2). Hypothesis testing on the three-molecular marker topology rejects the hypothesis that *Rhoicosphenia* is sister to *Gomphonema*, but does not reject the hypothesis that *Rhoicosphenia* is a member of the Heteroideae. The three-gene, SSU, and *rbc*L phylogenies also support the sister relationship of the Heteroideae and the Cymbellales + *Adlafia*. This is not a novel topology, as it has been evident in other molecular analyses [1, 38–40, 42], but has only been discussed in [42]. The only topology rejected by hypothesis testing on the three-molecular marker analysis was the sister relationship between *Rhoicosphenia* and *Gomphonema*. The Heteroideae were monophyletic in the three-molecular marker tree, so hypotheses H_2b,c_ were not tested and H_2a_ was not rejected (Table 4).

Analyses of concatenated alignments of two molecular markers generated three different topologies. The phylogeny based on SSU + LSU shows *Rhoicosphenia* as sister to *Anomoeoneis*, within the Cymbellales. This combination of molecular markers is the only one out of the seven molecular analyses to return this topology. It is interesting for two reasons. First, it is the only tree in which *Rhoicosphenia* is within, as opposed to outside the Cymbellales *sensu* Mann in [22]. Second, neither SSU nor LSU, when analyzed alone, return this result (S5 and S6 Figs). Although parts of the tree have low support, the node that places *Rhoicosphenia* within the Cymbellales has moderate support (83 BS, 97 BPP). Hypothesis testing only rejects the sister relationship between *Rhoicosphenia* and *Gomphonema*, and fails to reject the three different hypothesis in regards to the position of *Rhoicosphenia* relative to the Heteroideae.

SSU + *rbc*L, show a sister relationship between *Rhoicosphenia* and the two freshwater *Cocconeis* species. The clade including these taxa, along with the ‘monoraphid’ genera *Lemnicola* and *Achnanthidium* is sister to a clade of *Adlafia* + Cymbellales with moderate support (71 BS, 100 BPP). *Cocconeis stauroneiformis* is not sister to the ‘monoraphid’ genera, but is basal to the other Heteroideae + Cymbellales. Hypothesis H_2c_ was rejected, meaning that even though the most likely tree places *Rhoicosphenia* and the two freshwater *Cocconeis* species as sister taxa, this relationship has very low support. This alignment allowed the testing of all ‘monoraphid’ genera, including *Achnanthes sensu stricto*, and the monophyly of these genera was rejected, while the hypothesis of *Achnanthes sensu stricto* as sister to *Mastogloia* was not rejected.

LSU + *rbc*L recover a moderately-supported sister relationship between *Rhoicosphenia* and *Cocconeis* (76 BS, 98 BPP), and a less well-supported sister relationship between *Rhoicosphenia* + *Cocconeis* and *Achnanthidium* (45 BS, 98 BPP), the other ‘monoraphid’ taxon in the analysis. However, the sister relationship between the ‘monoraphid’ genera and Cymbellales is not supported in this analysis and *Cocconeis stauroneiformis* does not fall with the ‘monoraphid’ genera. Hypothesis testing rejected the hypothesis that *Rhoicosphenia* and *Gomphonema* are sister taxa.

The single molecular marker trees generated in this study supported different hypotheses of relationships for *Rhoicosphenia*. Other studies of diatoms analyzing multiple single molecular marker and concatenated alignments [38, 42, 55] demonstrate similar results, that is, not all single molecular marker trees recover the same tree topologies as each other or the concatenated alignment. Our single molecular marker analyses of SSU (8 BS) and *rbc*L (39 BS) suggest a weakly supported relationship between ‘monoraphid’ diatoms and *Rhoicosphenia*, together being sister to a moderately to poorly supported (SSU 63 BS, *rbc*L 26 BS) Cymbellales clade (S5 and S7 Figs). In the SSU analysis, *Rhoicosphenia* is sister to the Cymbellales clade with a branch support of 64 (ML bootstrap). Hypothesis testing could not reject *Rhoicosphenia* as either part of the Heteroideae, or as sister to *Gomphonema*. However, the hypothesis that all ‘monoraphid’ diatoms are monophyletic was rejected, while the hypothesis (H_6_) that *Achnanthes sensu stricto* is sister to *Mastogloia* was not rejected.

*rbc*L has weak support, 26 (ML BS), for a sister relationship between the Heteroideae and the Cymbellales, with *Rhoicosphenia* being sister to *Cocconeis* 39 (ML BS) deep within the Heteroideae. Hypothesis H_2c_ was rejected, meaning that even though the most likely trees places *Rhoicosphenia* and the two freshwater *Cocconeis* species as sister taxa, this relationship has very low support. Both the SSU and *rbc*L results support Mereschkowsky’s Pyrenophoreae [16], based on chloroplast number and structure but including diverse valve morphologies. Hypothesis testing of all ‘monoraphid’ diatoms, H_5_, was rejected with *rbc*L, however the hypothesis (H_6_) that *Achnanthes sensu stricto* is sister to *Mastogloia* was not rejected. Unlike SSU and *rbc*L, LSU places *Rhoicosphenia* sister to *Cocconeis* with weak support 34 (ML BS), with taxa not sister to the Cymbellales. However, deeper nodes in the LSU phylogram are very weakly supported <10 (ML BS), which could be reflective of LSU being a faster evolving marker in diatoms [79]. Our results with LSU and LSU + *rbc*L are similar to the LSU trees generated in [42, 55], in that their LSU returned the most unique topology of the three single molecular marker analyses. After analyzing all trees based on single, two-, and three-molecular markers we, similar to previous investigators [38, 40, 42, 55], have decided to base our conclusions on the three molecular marker concatenated alignment.

With regards to morphological analysis the strict consensus tree generated from 200 most parsimonious trees produced a large polytomy of taxa, with only congeneric species within the analysis being resolved together (S1 Fig). This result only differs from [50] (their Fig 5 and 6) in that their analysis groups some genera together, within a larger unresolved polytomy. This result, when compared to [50], indicates that our documentation and understanding of morphological characters that can inform a broad phylogeny of the raphid diatoms is currently insufficient.

In addition to the systematic position of *Rhoicosphenia*, our SSU analysis shows that the ‘monoraphid’ condition evolved multiple times, once in *Achnanthes sensu stricto*, and at least once in the other ‘monoraphid’ genera near the Cymbellales (S5 Fig), supporting hypotheses of Cleve [20] and Mereschkowsky [16]. Phylogenies showing this result have been returned in all analyses that include *Achnanthes sensu stricto* and other ‘monoraphid’ taxa [39, 40, 42, 52–55]. When considering morphology, the systematic position of *Achnanthes sensu stricto* is also quite interesting. Cox [51] suggested *Achnanthes* is closely related to *Mastogloia*, based on similarities in chloroplast, pore (cribrate), and raphe structure and cite their position in a cladistic analysis of morphology [50]. Our single molecular marker SSU, LSU and *rbc*L and multi-molecular marker analyses do not support a relationship between *Achnanthes* and *Mastogloia*, but instead place *Achnanthes* within the Bacillariales, similar to other molecular studies [42, 54, 55]. Mereschkowsky [16] showed the chloroplast of *Achnanthes sensu stricto* to be similar to *Hantzschia* Grunow [80], a genus within the Bacillariales. Placement of *Achnanthes* within the Bacillariales is problematic based on morphology, and more extensive taxon sampling in this region of the raphid diatom tree of life may help to resolve the phylogenetic position of this ‘monoraphid’ genus. Our molecular results, however, support the relationship between *Achnanthes* and the Bacillariales, but results of hypothesis testing do not rule out the possibility that *Achnanthes* is related to genera in the Mastogloiales. This appears to be another case, in addition to the relationships of ‘monoraphid’ diatoms and *Rhoicosphenia* with the Cymbellales, where molecular data support Mereschkowsky’s [16] suggestion of a close relationship between taxa with diverse valve morphologies, based on chloroplast similarities.

Since the description of *Rhoicosphenia* [2], multiple hypotheses of its phylogenetic position have been made based on valve [2] and chloroplast [16] morphology. Detailed investigations into the valve morphology [43], sexual reproduction [44], relation to other diatom genera [46], and initial cells and size reduction [45, 47] were unable to support or reject any of the hypotheses from the past century as summarized in [43], but did support Mann’s hypothesis (H_4_) that *Rhoicosphenia* belongs in an ‘enigmatic’ position [45]. Mann presented multiple lines of morphological evidence, without any formal analysis, that support the similarities of *Rhoicosphenia* to ‘monoraphid’ diatoms and *Gomphonema*, but explains their similarities as convergent evolution [43–45]. However, he did not question that the specific morphological traits he considers—pore occlusions, shape, heteropolarity, mucilage pads, pseudosepta, copulae, raphe structure and number, etc.–may look similar in different groups due to convergence (they are not homologous) and therefore would not be helpful in building phylogenies [43–45].

Based on the concatenated three molecular marker analysis, we suggest that *Rhoicosphenia* occupies a position basal to the Cymbellales. In terms of diatom classification, with the addition of the genera *Geissleria* [30, 31] and *Adlafia*, the Order Cymbellales *sensu* Round are a natural group—interestingly it is noted that *Adlafia* has a single chloroplast (as *Navicula brockmanii* Hustedt [81] in [42, 55]), similar to the chloroplast structure Mereschkowsky [16] used to unite the Monoplacatae, the group in which he placed members of the Cymbellales and *Rhoicosphenia*. While our data support Mereschkowsky’s Monoplacatae consisting of Heteroideae and Cymbellales, hypothesis testing rejects one specific proposal of Mereschkowsky, that is, the placement of *Rhoicosphenia* as sister to *Gomphonema* (Table 4). Our analysis supports the classification of [22] that places *Rhoicosphenia* in the Cymbellales, but we add phylogenetic structure to this grouping, with *Rhoicosphenia* in a basal position to the rest of the genera in the order. The order Cymbellales would now include the genera *Adlafia*, *Anomoeoneis*, *Cymbella*, *Cymbopleura*, *Didymosphenia*, *Encyonema*, *Encyonopsis*, *Geissleria*, *Gomphoneis*, *Gomphonema*, *Placoneis*, and *Reimeria*. The relationship between diatoms in the Heteroideae and the Cymbellales (including *Rhoicosphenia*) could be assigned a Linnaean taxonomic rank of superorder named Cymbellidae that would include Achnanthidiaceae + Cocconeidaceae + *Rhoicosphenia* + Cymbellales, within the subclass Bacillariophycidae. This superorder would be very similar to Mereschkowsky’s Monoplacatae, with the addition of genera that were not yet recognized in the early 20^th^ century, and would also represent a monophyletic clade in the context of PhyloCode [82]. The Cymbellales would remain an order in our classification, but two unnamed clades between the Order and Superorder ranks would also be recognized, one consisting of Cocconeidaceae + *Rhoicosphenia* + Cymbellales, the other would consist of *Rhoicosphenia* + Cymbellales. Additionally, our results support Mereschkowsky [16] and Cox [51] that *Achnanthes sensu stricto* should not be considered part of a monophyletic clade of ‘monoraphid’ diatoms, however cannot fully support or reject their specific placements of the genus. Finally, our analyses support Cleve’s [20] hypothesis that ‘monoraphid’ diatoms are polyphyletic. A classification scheme based on our results is presented below.

- SUPERORDER: Cymbellidae (Achnanthidiaceae + Cocconeidaceae + *Rhoicosphenia* + Cymbellales)
  - Unnamed Clade (Cocconeidaceae + *Rhoicosphenia* + Cymbellales)
    - Unnamed Clade (*Rhoicosphenia* + Cymbellales)
      - ORDER: Cymbellales (*Adlafia*, *Anomoeoneis*, *Cymbella*, *Cymbopleura*, *Didymosphenia*, *Encyonema*, *Encyonopsis*, *Geissleria*, *Gomphoneis*, *Gomphonema*, *Placoneis*, *Reimeria*, *Rhoicosphenia*)
        - Suborder: **Cymbellineae**, Suborder nov.
          - Family: Cymbellaceae Grunow (*Adlafia*, *Anomoeoneis*, *Cymbella*, *Cymbopleura*, *Didymosphenia*, *Encyonema*, *Encyonopsis*, *Geissleria*, *Gomphoneis*, *Gomphonema*, *Placoneis*, *Reimeria*)

## Supporting Information

S1 File SSU + LSU + *rbc*L alignment. Alignment used in Maximum Likelihood and Bayesian analyses.
(FASTA)

S2 File SSU + LSU alignment. Alignment used in Maximum Likelihood and Bayesian analyses.
(FASTA)

S3 File SSU + *rbc*L alignment. Alignment used in Maximum Likelihood and Bayesian analyses.
(FASTA)

S4 File LSU + *rbc*L alignment. Alignment used in Maximum Likelihood and Bayesian analyses.
(FASTA)

S5 File SSU alignment. Alignment used in Maximum Likelihood and Bayesian analyses.
(FASTA)

S6 File LSU alignment. Alignment used in Maximum Likelihood and Bayesian analyses.
(FASTA)

S7 File *rbc*L alignment. Alignment used in Maximum Likelihood and Bayesian analyses.
(FASTA)

S1 Fig Strict consensus tree of morphological characters. Resulting phylogram of morphological analysis.
(PDF)

S2 Fig SSU + LSU Maximum Likelihood (a) and Bayesian (b) phylograms. Node support values for (a) are maximum likelihood bootstrap values (500 bootstraps), and (b) are Bayesian posterior probability (as a percentage).
(PDF)

S3 Fig SSU + *rbc*L Maximum Likelihood (a) and Bayesian (b) phylograms. Node support values for (a) are maximum likelihood bootstrap values (500 bootstraps), and (b) are Bayesian posterior probability (as a percentage).
(PDF)

S4 Fig LSU + *rbc*L Maximum Likelihood (a) and Bayesian (b) phylograms. Node support values for (a) are maximum likelihood bootstrap values (500 bootstraps), and (b) are Bayesian posterior probability (as a percentage).
(PDF)

S5 Fig SSU Maximum Likelihood (a) and Bayesian (b) phylograms. Node support values for (a) are maximum likelihood bootstrap values (500 bootstraps), and (b) are Bayesian posterior probability (as a percentage).
(PDF)

S6 Fig LSU Maximum Likelihood (a) and Bayesian (b) phylograms. Node support values for (a) are maximum likelihood bootstrap values (500 bootstraps), and (b) are Bayesian posterior probability (as a percentage).
(PDF)

S7 Fig *rbc*L Maximum Likelihood (a) and Bayesian (b) phylograms. Node support values for (a) are maximum likelihood bootstrap values (500 bootstraps), and (b) are Bayesian posterior probability (as a percentage).
(PDF)

S1 Table Sequences used for three single marker analyses. List of taxa used in single marker analyses for SSU, LSU, and *rbc*L, where sequence is used, its GenBank Accession number is present in corresponding cell.
(DOCX)

S2 Table Sequences included in three-marker concatenated alignment. Taxa and GenBank Accession numbers that were included in three-marker (SSU, LSU, *rbc*L) alignment.
(DOCX)

S3 Table Characters and character states. Characters and character states used in morphological phylogenetic analysis.
(PDF)

S4 Table Taxon and character matrix. Taxa and corresponding characters used in morphological phylogenetic analysis.
(PDF)
