# Supplementary figures and images for: Historical and Current Perspectives on the Systematics of the ‘Enigmatic’ Diatom Genus Rhoicosphenia (Bacillariophyta), with Single and Multi-Molecular Marker and Morphological Analyses and Discussion on the Monophyly of ‘Monoraphid’ Diatoms
Source: PLoS One. 2016 Apr 5;11(4):e0152797. doi: 10.1371/journal.pone.0152797 (PMC4821588; doi:10.1371/journal.pone.0152797)

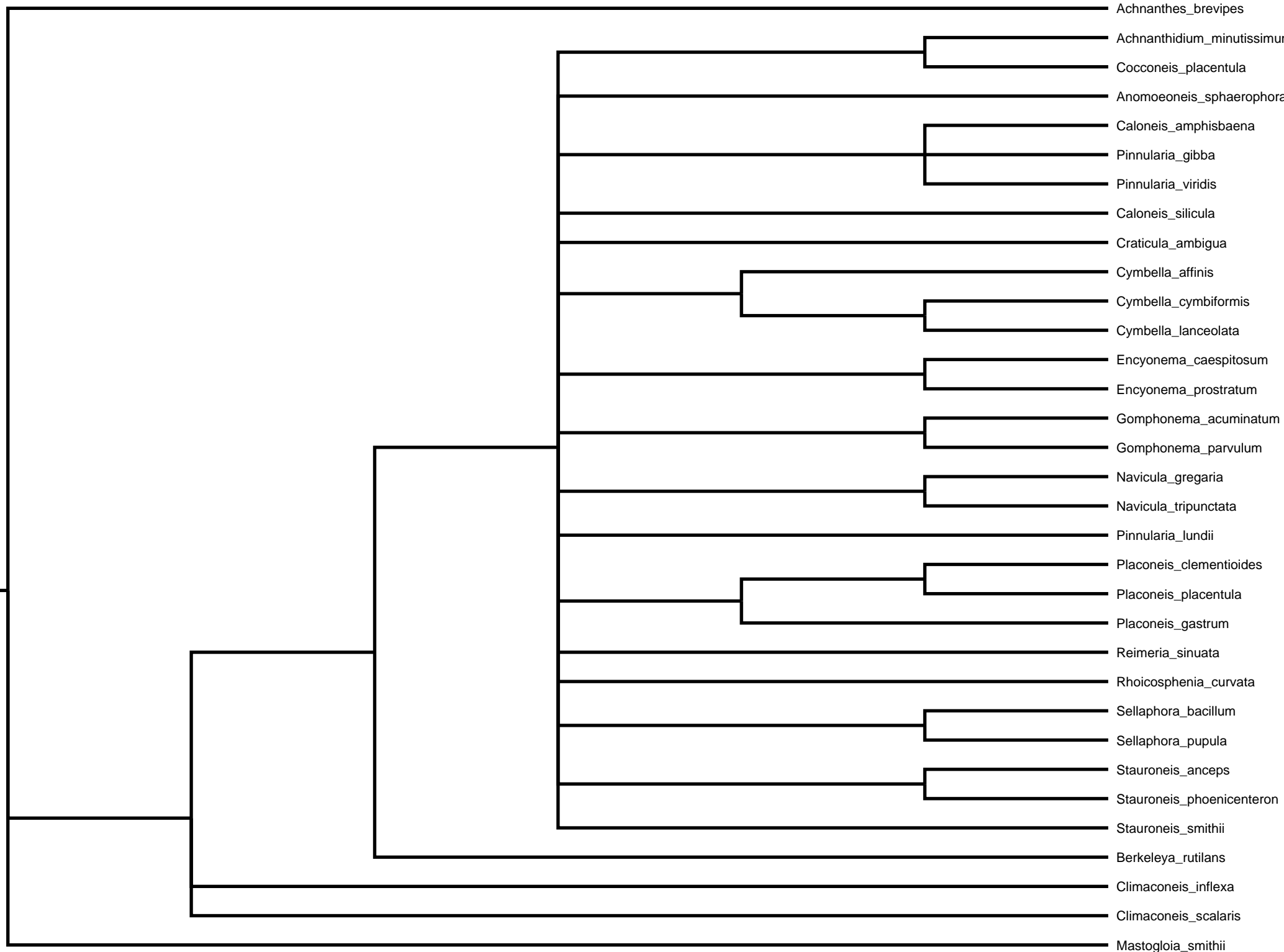

0.7

Supplement: S1 Fig — Resulting phylogram of morphological analysis. (PDF) [file pone.0152797.s008.pdf]

a)

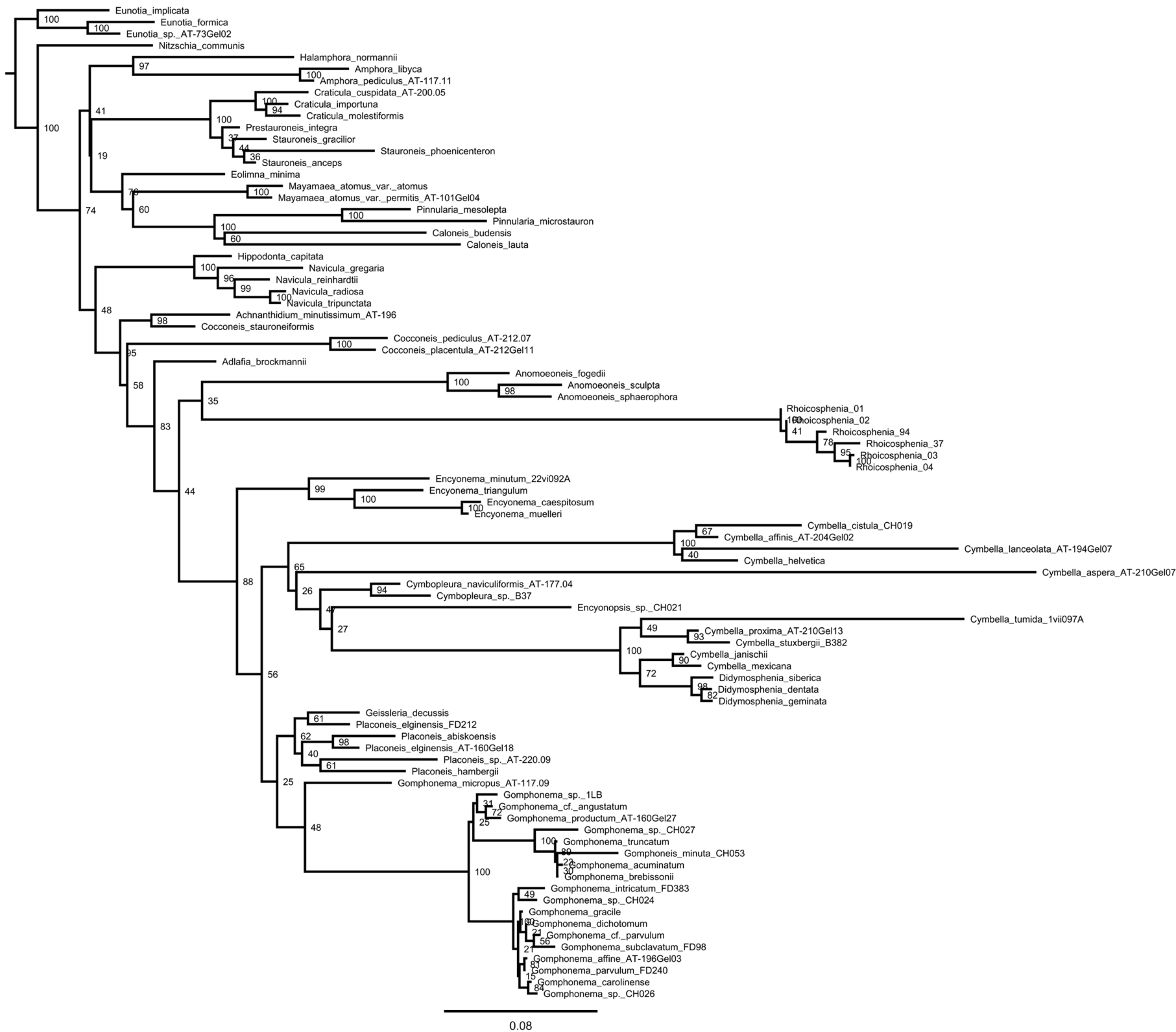

b)

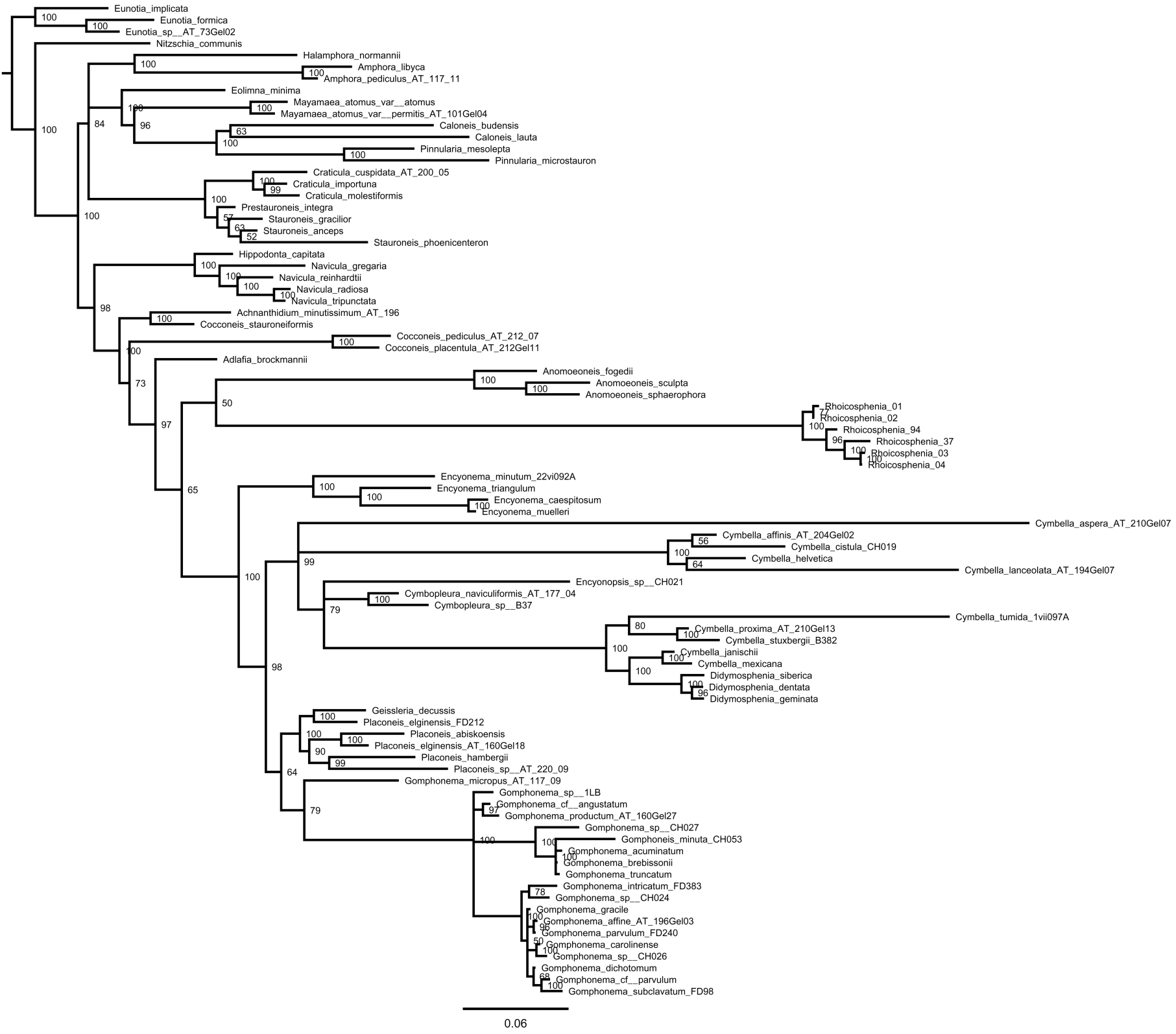

Supplement: S2 Fig — Node support values for (a) are maximum likelihood bootstrap values (500 bootstraps), and (b) are Bayesian posterior probability (as a percentage). (PDF) [file pone.0152797.s009.pdf]

a)

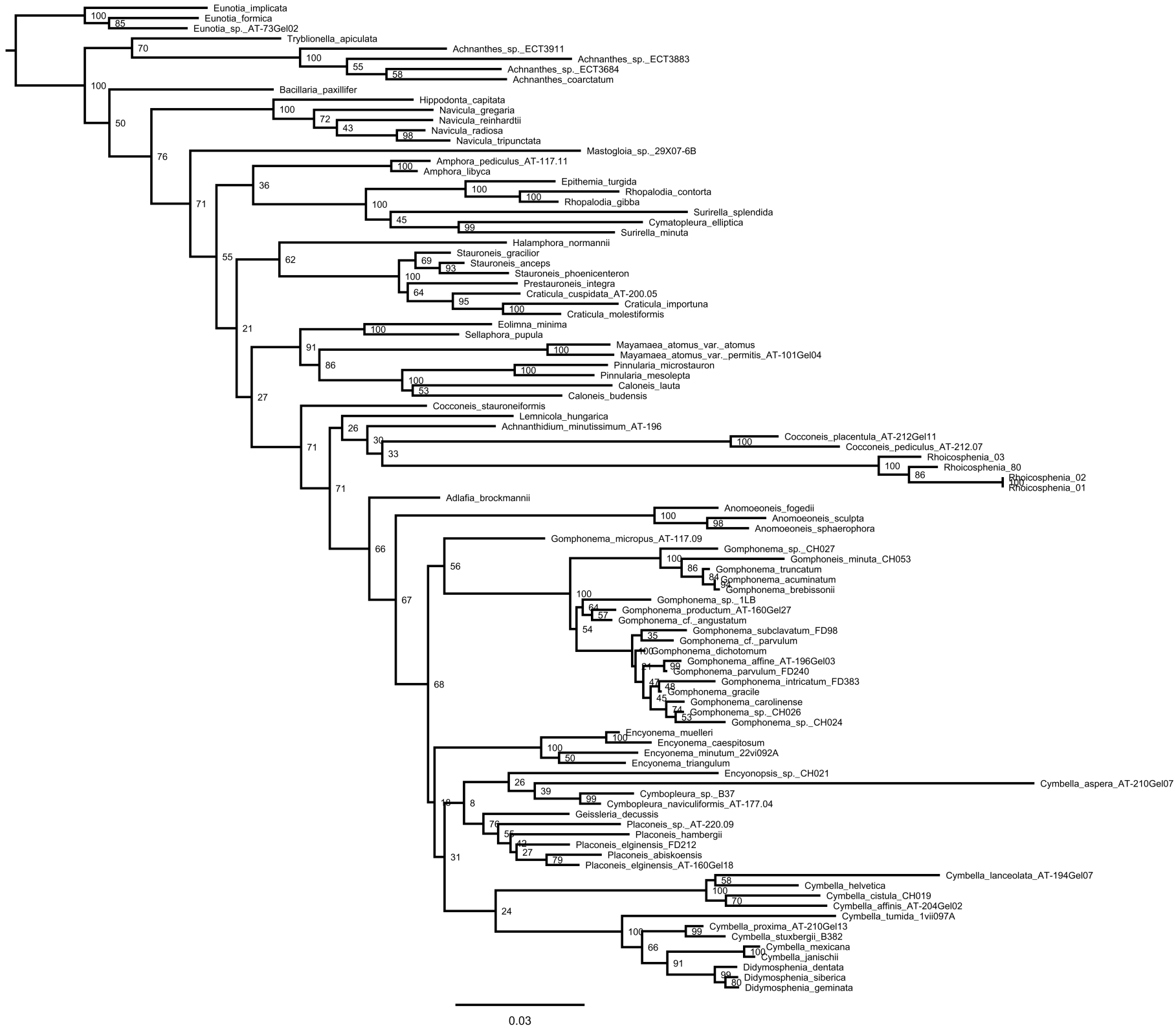

b)

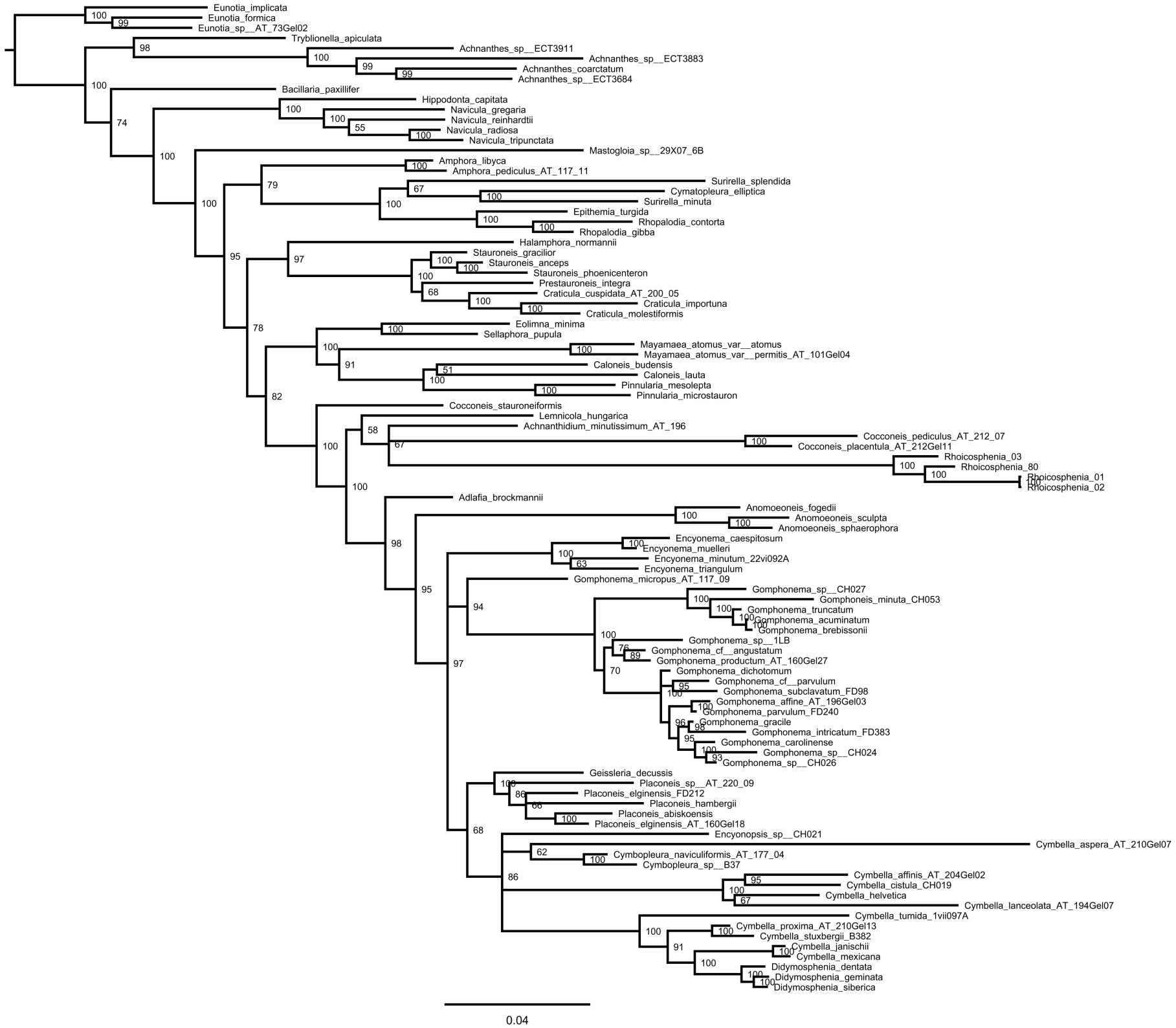

Supplement: S3 Fig — Node support values for (a) are maximum likelihood bootstrap values (500 bootstraps), and (b) are Bayesian posterior probability (as a percentage). (PDF) [file pone.0152797.s010.pdf]

a)

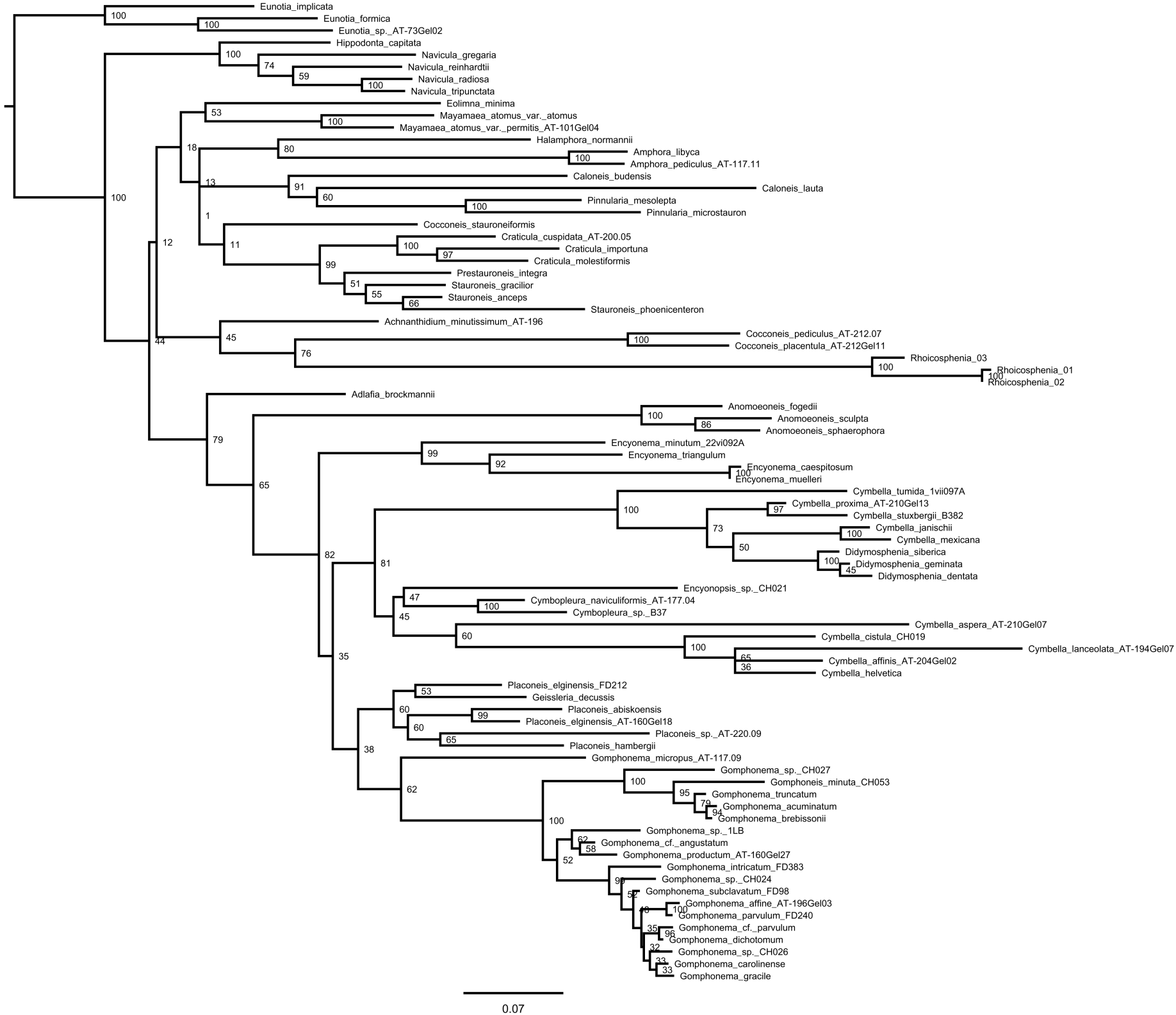

b)

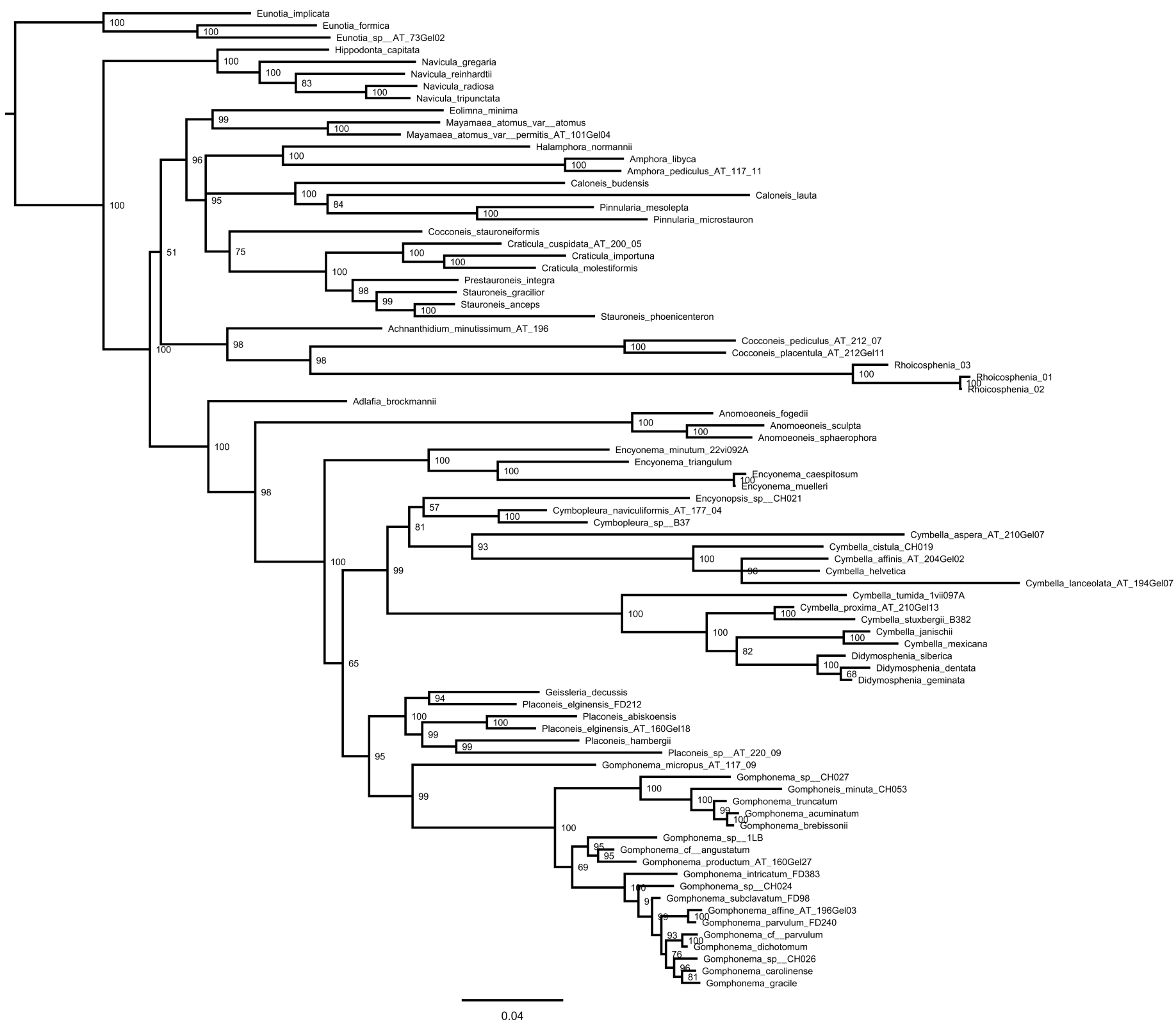

Supplement: S4 Fig — Node support values for (a) are maximum likelihood bootstrap values (500 bootstraps), and (b) are Bayesian posterior probability (as a percentage). (PDF) [file pone.0152797.s011.pdf]

a)

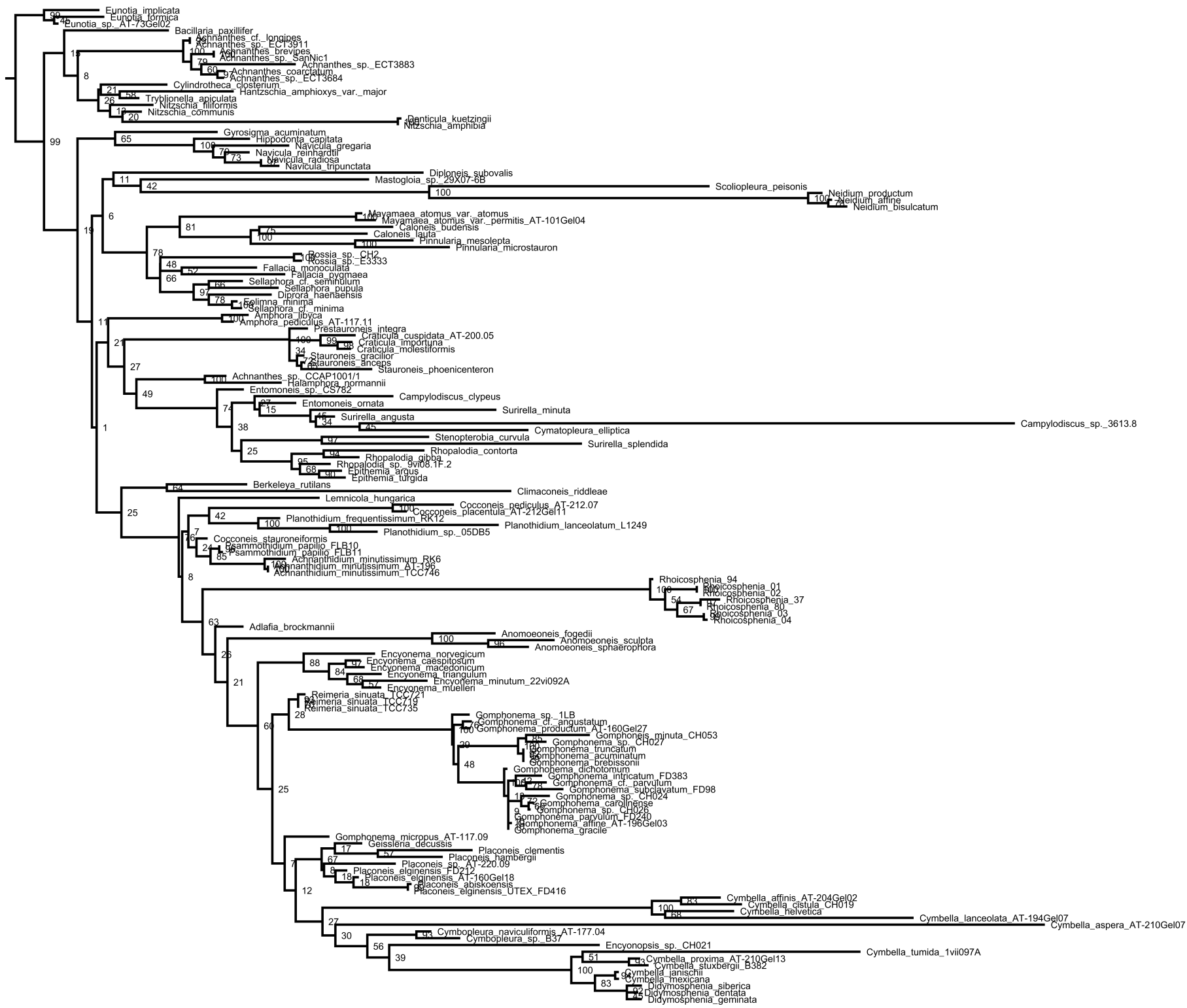

b)

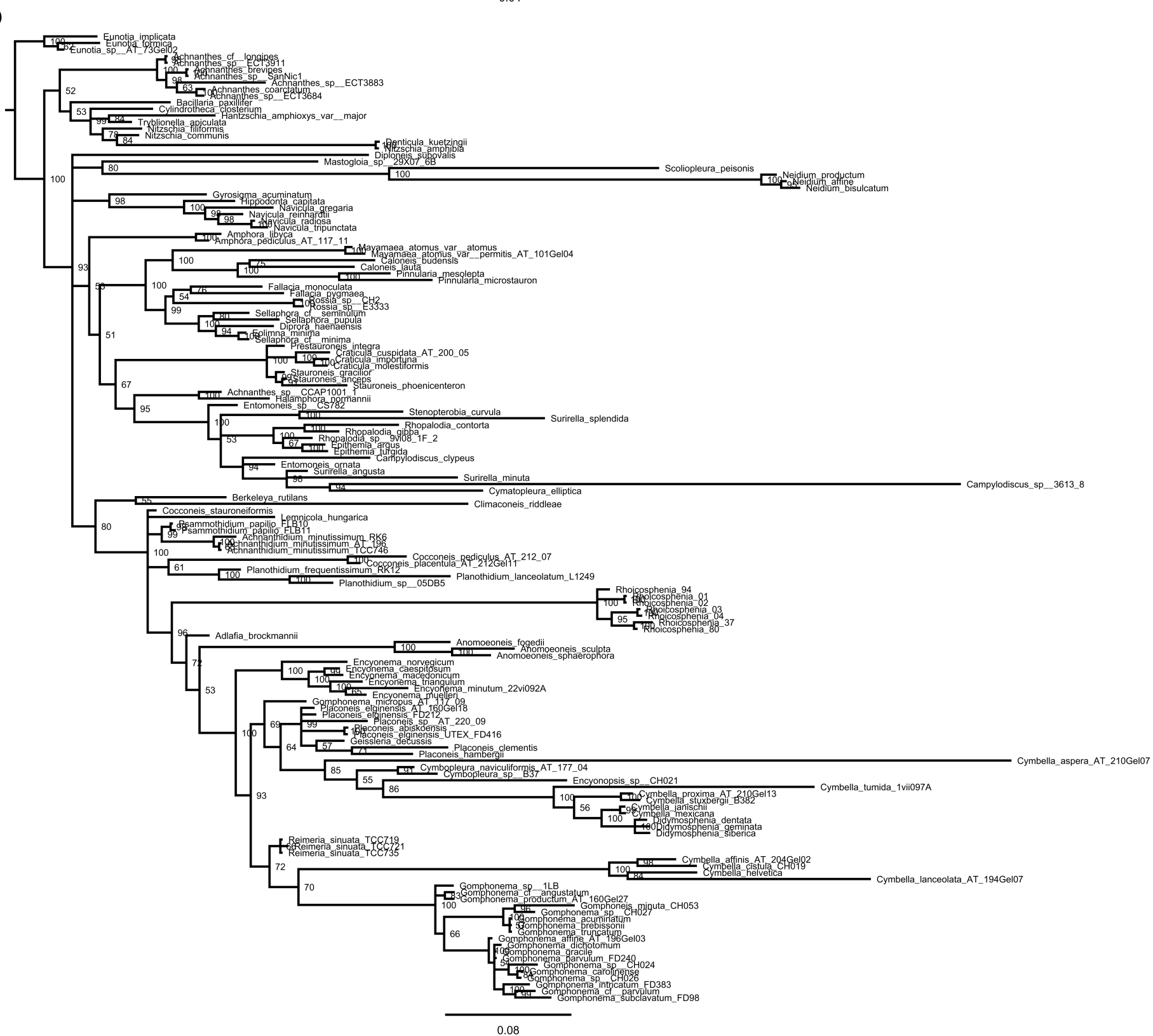

Supplement: S5 Fig — Node support values for (a) are maximum likelihood bootstrap values (500 bootstraps), and (b) are Bayesian posterior probability (as a percentage). (PDF) [file pone.0152797.s012.pdf]

a)

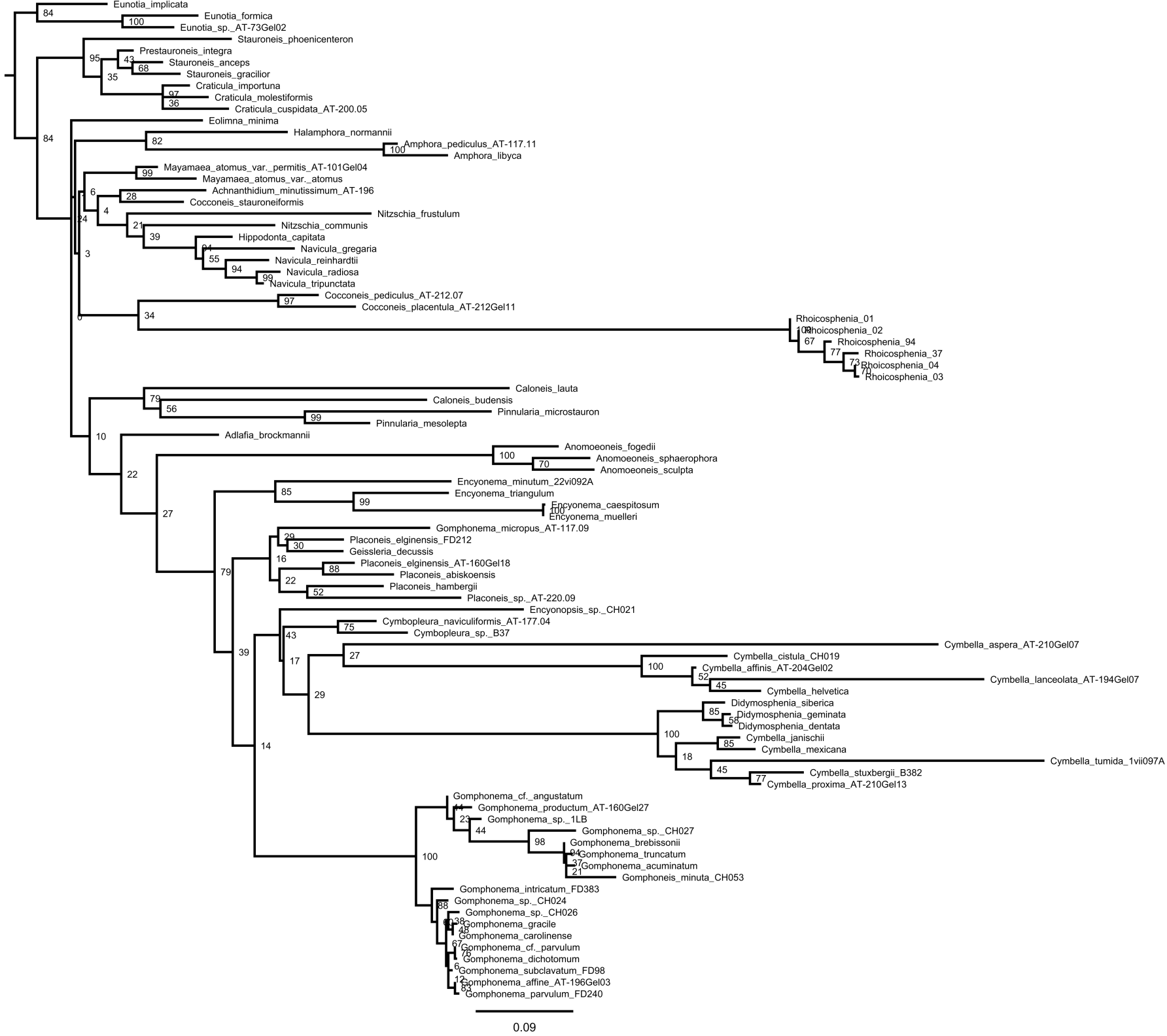

b)

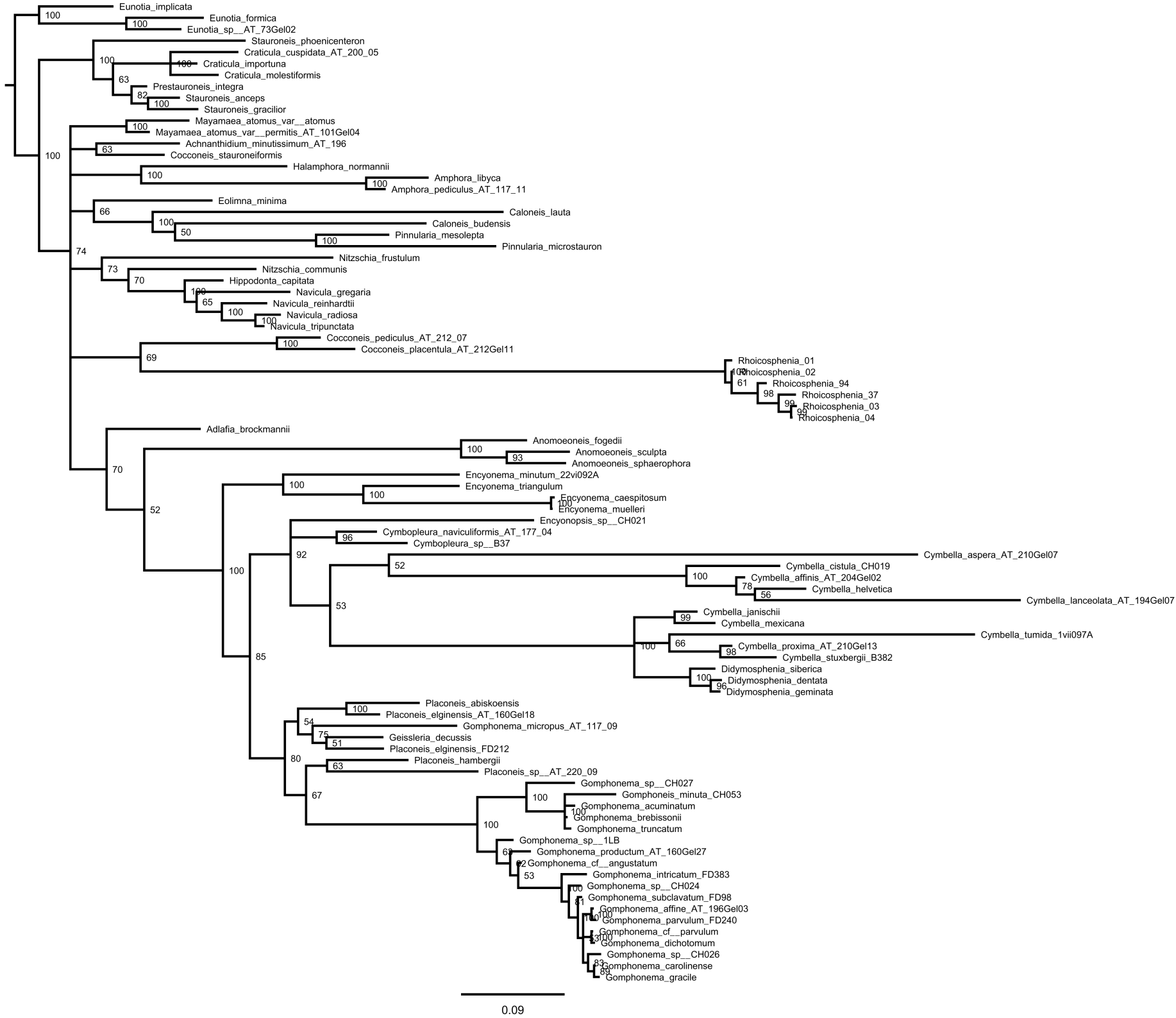

Supplement: S6 Fig — Node support values for (a) are maximum likelihood bootstrap values (500 bootstraps), and (b) are Bayesian posterior probability (as a percentage). (PDF) [file pone.0152797.s013.pdf]

a)

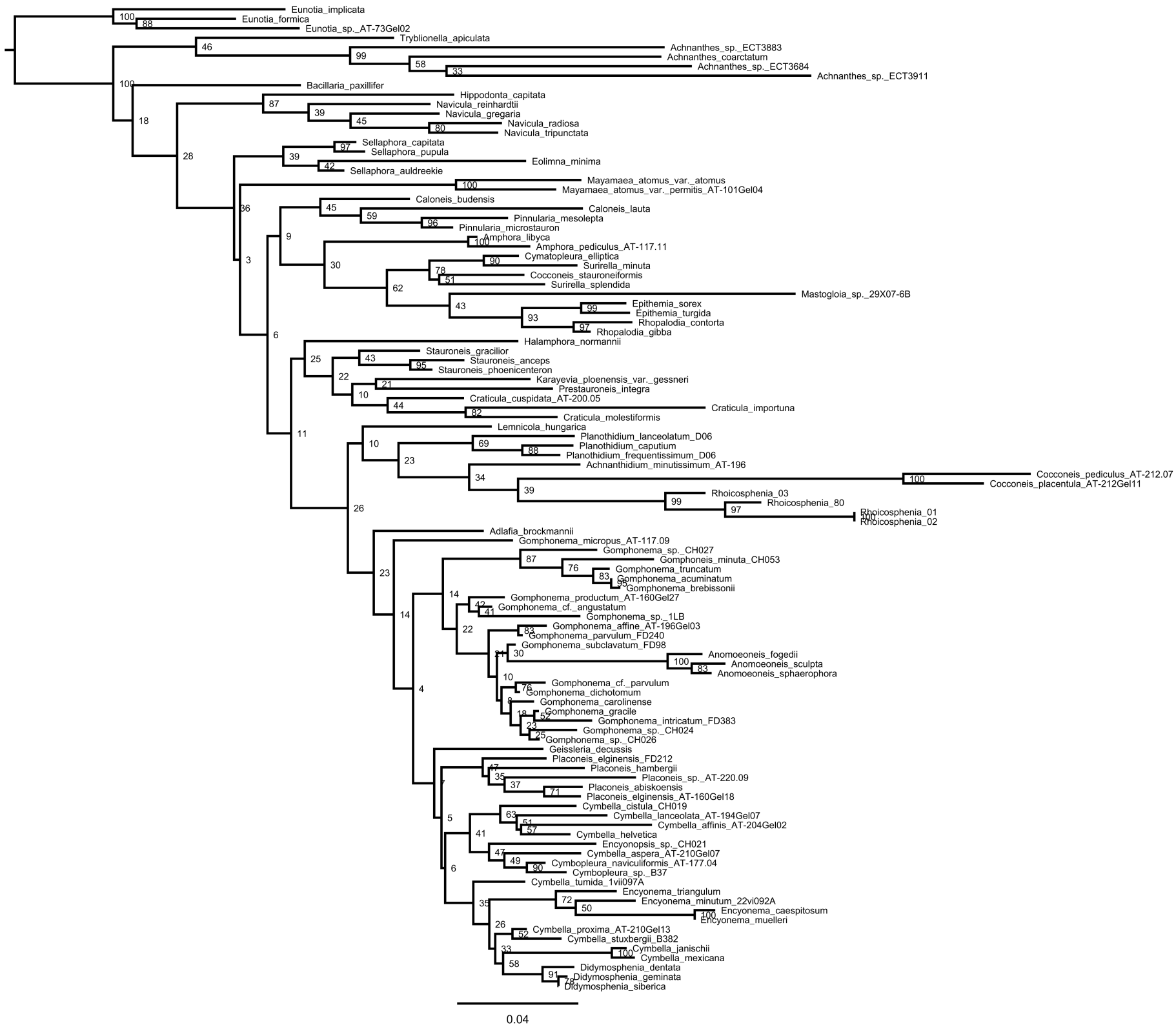

b)

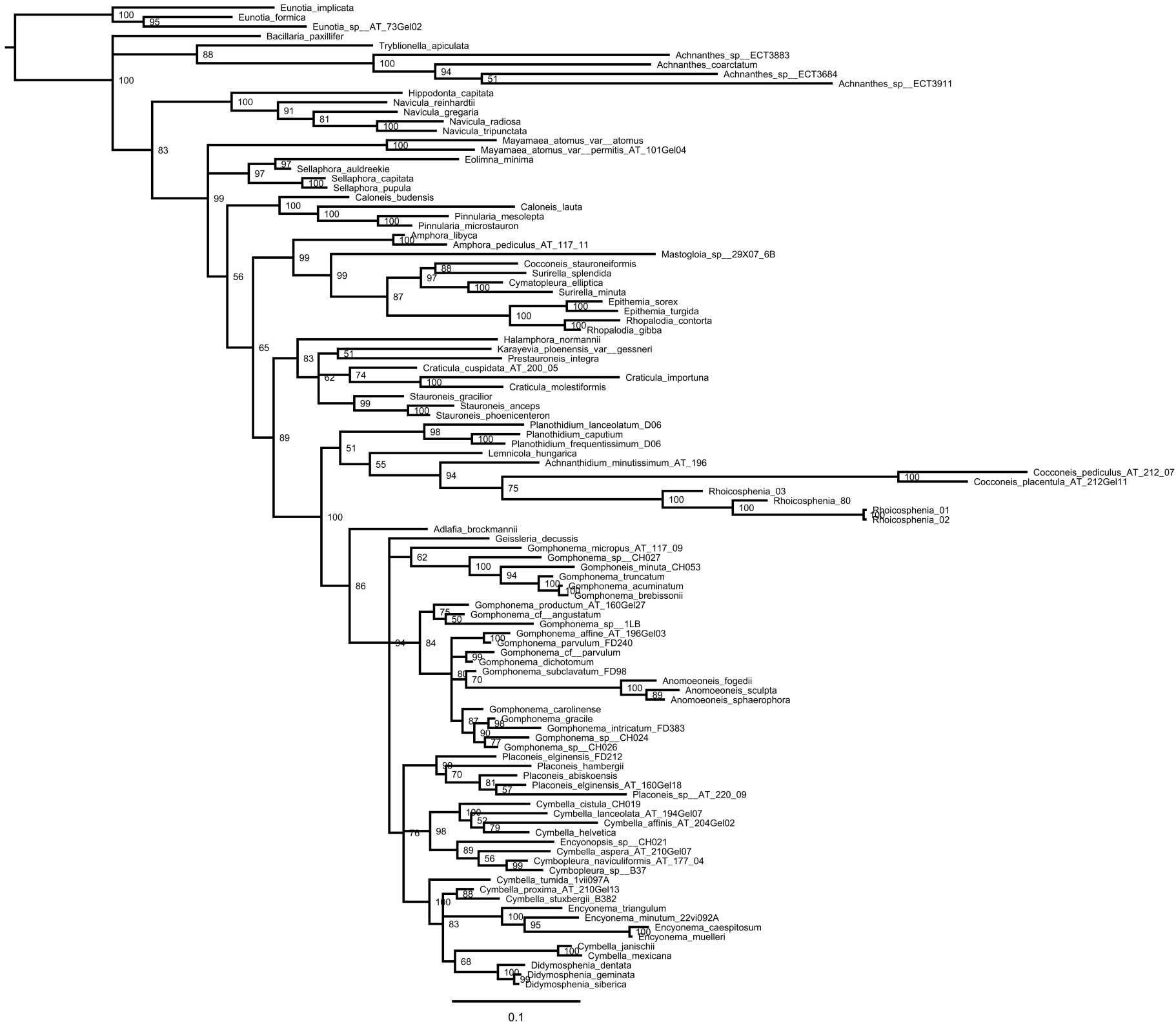

Supplement: S7 Fig — Node support values for (a) are maximum likelihood bootstrap values (500 bootstraps), and (b) are Bayesian posterior probability (as a percentage). (PDF) [file pone.0152797.s014.pdf]
