# Supplementary material for: Historical and Current Perspectives on the Systematics of the ‘Enigmatic’ Diatom Genus Rhoicosphenia (Bacillariophyta), with Single and Multi-Molecular Marker and Morphological Analyses and Discussion on the Monophyly of ‘Monoraphid’ Diatoms
Source: PLoS One. 2016 Apr 5;11(4):e0152797. doi: 10.1371/journal.pone.0152797 (PMC4821588; doi:10.1371/journal.pone.0152797)
Supplement: S1 Table — List of taxa used in single marker analyses for SSU, LSU, and rbcL, where sequence is used, its GenBank Accession number is present in corresponding cell. (DOCX) [file pone.0152797.s015.docx]

**S1 Table:** GenBank sequences used in analyses. An accession number below the molecular marker name means that particular taxon was used in that analysis.

| **Full name with Authority** | **Culture ID** | **SSU** | **LSU** | ***rbc*L** |
| --- | --- | --- | --- | --- |
| *Achnanthes* *brevipes* Agardh | CCMP100 | AY485476 |  |  |
| *Achnanthes* cf. *longipes* Agardh | CCMP101 | AY485500.1 |  |  |
| *Achnanthes* *coarctata* (Brébisson ex W. Smith) Grunow in Cleve & Grunow | FD185 | HQ912594.1 |  | HQ912458.1 |
| *Achnanthes* sp. | CCAP1001/1 | AY485496 |  |  |
| *Achnanthes* sp. 1 | ECT3684 | KC309476 |  | KC309548.1 |
| *Achnanthes* sp. 1 | ECT3883 | KC309474.1 |  | KC309546.1 |
| *Achnanthes* sp. 1 | ECT3911 | KC309475.1 |  | KC309547.1 |
| *Achnanthes* sp. 1 | SanNic1 | KC309473.1 |  |  |
| *Achnanthidium* *minutissimum* (Kützing) Czarnecki | AT-196Gel02 | AM502032 | AM710588 | AM710499 |
| *Achnanthidium* *minutissimum* (Kützing) Czarnecki | RK6 | KF417666.1 |  |  |
| *Achnanthidium* *minutissimum* (Kützing) Czarnecki | TCC746 | KF959663.1 |  |  |
| *Adlafia* *brockmannii* (Hustedt) K. Bruder in K. Bruder & L.K. Medlin | AT-111Gel10 | AM502020 | AM710576 | AM710487 |
| *Amphora* *libyca* Ehrenberg | AT-117.10 | AM501959 | AM710513 | AM710425 |
| *Amphora* *pediculus* (Kützing) Grunow in Schmidt *et al.* | AT-117.11 | AM501960 | AM710514 | AM710426 |
| *Anomoeoneis* *fogedii* Reimer | FD399 | KJ011610 | KJ011555 | KJ011793 |
| *Anomoeoneis* *sculpta* (Ehrenberg) Cleve | CH239 | KJ011611 | KJ011556 | KJ011794 |
| *Anomoeoneis* *sphaerophora* Pfitzer | FD160 | KJ011612 | KJ011557 | KJ011795 |
| *Bacillaria* *paxillifer* (O. Müller) Hendey | FD468 | HQ912627 |  | HQ912491 |
| *Berkeleya* *rutilans* (Trentepohl ex Roth) Grunow | ECT3616 | HQ912637 |  |  |
| *Caloneis* *budensis* (Grunow) Krammer in Krammer & Lange-Bertalot | AT-220.06 | AM502003 | AM710559 | AM710470 |
| *Caloneis* *lauta* Carter & Bailey-Watts | AT-160Gel04 | AM502039 | AM710595 | AM710506 |
| *Campylodiscus* *clypeus* Ehrenberg | L951 | HQ912412 |  |  |
| *Campylodiscus* sp. | 3613.8 | HQ912413 |  |  |
| *Climaconeis* *riddleae* A.K.S. Prasad | ECT3724 | HQ912644 |  |  |
| *Cocconeis* *pediculus* Ehrenberg | AT-212.07 | AM502010 | AM710566 | AM710477 |
| *Cocconeis* *placentula* Ehrenberg | AT-212Gel11 | AM502013 | AM710569 | AM710480 |
| *Cocconeis* *stauroneiformis* (W. Smith; Rabenhorst) Okuno | S0230 | AB430614.1 | AB430654.1 | AB430694.1 |
| *Craticula* *cuspidata* (Kützing) Mann | AT-200.05 | AM501998 | AM710554 | AM710465 |
| *Craticula* *importuna* (Hustedt) K. Bruder & Hinz in K. Bruder & L.K. Medlin | AT-70Gel14a | AM501978 | AM710533 | AM710444 |
| *Craticula* *molestiformis* (Hustedt) Mayama | AT-36.klein | AM501989 | AM710532 | AM710455 |
| *Cylindrotheca* *closterium* (Ehrenberg) Reimann & Lewin | CCMP1855 | HQ912645 |  |  |
| *Cymatopleura* *elliptica* (Brebisson ex Kützing) W. Smith | L1333 | HQ912659 |  | HQ912523 |
| *Cymbella* *affinis* Kützing | AT-204Gel02 | AM502009 | AM710565 | AM710476 |
| *Cymbella* *aspera* (Ehrenberg) Cleve | AT-210Gel07 | AM502016 | AM710572 | AM710483 |
| *Cymbella* *cistula* (Hemprich in Hemprich & Ehrenberg) Kirchner | CH019 | KJ011618 | KJ011562 | KJ011801 |
| *Cymbella* *helvetica* Kützing | B457 | KJ011621 | KJ011565 | KJ011804 |
| *Cymbella* *janischii* (Schmidt in Schmidt *et al.*) Cleve | CH062 | KJ011622 | KJ011566 | KJ011805 |
| *Cymbella* *lanceolata* (Agardh) Kirchner | AT-194Gel07 | AM502026 | AM710582 | AM710493 |
| *Cymbella* *mexicana* (Ehrenberg) Cleve | CH031 | KJ011624 | KJ011568 | KJ011807 |
| *Cymbella* *proxima* Reimer in Patrick & Reimer | AT-210Gel13 | AM502017 | AM710573 | AM710484 |
| *Cymbella* *stuxbergii* (Cleve) Cleve | B382 | KJ011628 | KJ011572 | KJ011811 |
| *Cymbella* *tumida* (Brebisson ex Kützing) Van Heurck | 1vii097A | KJ011629 | KJ011573 | KJ011812 |
| *Cymbopleura* *naviculiformis* (Auerswald ex Heiberg) Krammer | AT-177.04 | AM501997 | AM710553 | AM710464 |
| *Cymbopleura* sp. | TN-2014 B37 | KJ011633 | KJ011577 | KJ011816 |
| *Didymosphenia* *dentata* (Dorogostaisky) Skvortzow | B547 | KJ011635 | KJ011579 | KJ011818 |
| *Didymosphenia* *geminata* (Lyngbye) M. Schmidt in Schmidt *et al.* | CH058 | KJ011636 | KJ011580 | KJ011819 |
| *Didymosphenia* *siberica* (Grunow) M. Schmidt | B40 | KJ011637 | KJ011581 | KJ011820 |
| *Diploneis* *subovalis* Cleve | FD282 | HQ912597 |  |  |
| *Diprora* *haenaensis* Main | 8296-Dipr001 | KC954571 |  |  |
| *Encyonema* *caespitosum* Kützing | AT-214Gel03 | AM502035 | AM710591 | AM710502 |
| *Encyonema* *macedonicum* Levkov, Metzeltin & Krstic | CH011 | KJ011638 |  |  |
| *Encyonema* *minutum* (Hilse in Rabenhorst) Mann in Round, Crawford & Mann | 22vi092A | KJ011640 | KJ011582 | KJ011823 |
| *Encyonema* *muelleri* (Hustedt) Mann in Round, Crawford & Mann | 16vi091B | KJ011642 | KJ011584 | KJ011825 |
| *Encyonema* *norvegicum* (Grunow) Mayer | FD342 | KJ011643 |  |  |
| *Encyonema* *triangulum* (Ehrenberg) Kützing | 2vii091 | KJ011645 | KJ011586 | KJ011828 |
| *Encyonopsis* sp. | CH021 | KJ011646 | KJ011587 | KJ011829 |
| *Entomoneis* *ornata* (Bailey) Reimer in Patrick & Reimer | 14A | HQ912411 |  |  |
| *Entomoneis* sp. | CS782 | HQ912631 |  |  |
| *Eolimna* *minima* (Grunow in Van Heurck) Lange-Bertalot | AT-70Gel18 | AM501962 | AM710516 | AM710427 |
| *Epithemia* *argus* (Ehrenberg) Kützing | CH211 | HQ912408 |  |  |
| *Epithemia* *turgida* (Ehrenberg) Kützing | CH154 | HQ912410 |  | HQ912396 |
| *Eunotia* *formica* Ehrenberg | AT-111Gel09 | AM502040 | AM710517 | AM710428 |
| *Eunotia* *implicata* Norpel, Alles & Lange-Bertalot | AT-219.07 | AM502001 | AM710557 | AM710468 |
| *Eunotia* sp. | AT-73Gel02 | AM501963 | AM710518 | AM710429 |
| *Fallacia* *monoculata* (Hustedt) Mann | FD254 | HQ912596 |  |  |
| *Fallacia* *pygmaea* (Kützing) Stickle & Mann | FD294 | HQ912605 |  |  |
| *Geissleria* *decussis* (Østrup) Lange-Bertalot & Metzeltin | FD50 | KJ011647 | KJ011588 | KJ011830 |
| *Gomphoneis* *minuta* (Stone) Kociolek & Stoermer | CH053 | KJ011648 | KJ011589 | KJ011831 |
| *Gomphonema* *acuminatum* Ehrenberg | AT-219Gel10 | AM502019 | AM710575 | AM710486 |
| *Gomphonema* *affine* (Gregory) Andrews | AT-196Gel03 | AM502033 | AM710589 | AM710500 |
| *Gomphonema* *brebissonii* Kützing | FD373 | KJ011653 | KJ011593 | KJ011836 |
| *Gomphonema* *carolinense* Hagelstein | FD285 | KJ011654 | KJ011594 | KJ011837 |
| *Gomphonema* cf. *angustatum* (Kützing) Rabenhorst | AT-109Gel08b | AM502005 | AM710561 | AM710472 |
| *Gomphonema* cf. *parvulum* (Kützing) Kützing | AT-161.15 | AM501995 | AM710551 | AM710462 |
| *Gomphonema* *dichotomum* Kützing | FD288 | KJ011655 | KJ011595 | KJ011838 |
| *Gomphonema* *gracile* Ehrenberg | FD65 | KJ011656 | KJ011596 | KJ011839 |
| *Gomphonema* *intricatum* Kützing | FD383 | KJ011658 | KJ011598 | KJ011841 |
| *Gomphonema* *micropus* Kützing | AT-117.09 | AM501964 | AM710519 | AM710430 |
| *Gomphonema* *parvulum* (Kützing) Kützing | FD240 | KJ011659 | KJ011599 | KJ011842 |
| *Gomphonema* *productum* (Grunow) Lange-Bertalot & Reichardt | AT-160Gel27 | AM501993 | AM710549 | AM710460 |
| *Gomphonema* sp. | CH024 | KJ011662 | KJ011602 | KJ011845 |
| *Gomphonema* sp. | CH026 | KJ011663 | KJ011603 | KJ011846 |
| *Gomphonema* sp. | CH027 | KJ011664 | KJ011604 | KJ011847 |
| *Gomphonema* sp. 1LB | B559 | KJ011660 | KJ011600 | KJ011843 |
| *Gomphonema* *subclavatum* var. *commutatum* (Grunow) Mayer | FD98 | KJ011665 | KJ011605 | KJ011848 |
| *Gomphonema* *truncatum* Ehrenberg | AT-195Gel09 | AM501956 | AM710598 | AM710509 |
| *Gyrosigma* *acuminatum* (Kützing) Rabenhorst | FD317 | HQ912598 |  |  |
| *Halamphora* *normannii* (Rabenhorst) Z. Levkov | AT-105Gel05 | AM501958 | AM710512 | AM710424 |
| *Hantzschia* *amphioxys* var. *major* Grunow in Van Heurck | A4 | HQ912404 |  |  |
| *Hippodonta* *capitata* (Ehrenberg) Lange-Bertalot, Metzeltin & Witkowski | AT-124.24 | AM501966 | AM710521 | AM710432 |
| *Lemnicola* *hungarica* (Grunow) Round & Basson | FD456 | HQ912626.1 |  | HQ912490.1 |
| *Mastogloia* sp. | 29X07-6B | HQ912632 |  | HQ912496 |
| *Mayamaea* *atomus* var. *atomus* (Kützing) Lange-Bertalot | AT-115Gel07 | AM501968 | AM710523 | AM710434 |
| *Mayamaea* *atomus* var. *permitis* (Hustedt) Lange-Bertalot | AT-101Gel04 | AM501969 | AM710524 | AM710435 |
| *Navicula* *gregaria* Donkin | AT-117Gel05 | AM501974 | AM710529 | AM710440 |
| *Navicula* *radiosa* Kützing | AT-114Gel06 | AM502034 | AM710527 | AM710501 |
| *Navicula* *reinhardtii* (Grunow) Grunow | AT-124.15 | AM501976 | AM710531 | AM710442 |
| *Navicula* *tripunctata* (O.F. Müller) Bory | AT-202.01 | AM502028 | AM710584 | AM710495 |
| *Neidium* *affine* (Ehrenberg) Pfitzer | FD127 | HQ912583 |  |  |
| *Neidium* *bisulcatum* var. *subampliatum* Krammer | FD417 | HQ912591 |  |  |
| *Neidium* *productum* (W. Smith) Cleve | FD116 | HQ912582 |  |  |
| *Nitzschia* *amphibia* Grunow | FDCC L602 | AJ867277 |  |  |
| *Nitzschia* *communis* Rabenhorst | FDCC L408 | AJ867278 | AF417661 |  |
| *Nitzschia* *filiformis* (W. Smith) Van Heurck | FD267 | HQ912589 |  |  |
| *Pinnularia* *mesolepta* (Ehrenberg) W. Smith | AT-160Gel30 | AM501994 | AM710550 | AM710461 |
| *Pinnularia* *microstauron* (Ehrenberg) Cleve | AT-105Gel08 | AM501981 | AM710536 | AM710447 |
| *Placoneis* *abiskoensis* (Hustedt) Lange-Bertalot & Metzeltin | FD363 | KJ011667 | KJ011607 | KJ011850 |
| *Placoneis* *clementis* (Grunow) Cox | FD419 | KJ011668 |  |  |
| *Placoneis* *elginensis* (Gregory) Cox | AT-160Gel18 | AM501953 | AM710548 | AM710459 |
| *Placoneis* *elginensis* (Gregory) Cox | FD212 | KJ011669 | KJ011608 | KJ011852 |
| *Placoneis* *elginensis* (Gregory) Cox | FD416 | HQ912607 |  |  |
| *Placoneis* *hambergii* (Hustedt) K. Bruder | AT-160Gel09 | AM502030 | AM710586 | AM710497 |
| *Placoneis* sp. | AT-220.09 | AM502014 | AM710570 | AM710481 |
| *Planothidium* *frequentissimum* (Lange-Bertalot) Round & Bukhtiyarova | RK12 | KF417663.1 |  |  |
| *Planothidium* *lanceolatum* (Brébisson ex Kützing) Lange-Bertalot | L1249 | AJ535189.1 |  |  |
| *Planothidium* sp. | 05DB5 | KF417664.1 |  |  |
| *Prestauroneis* *integra* (W. Smith) K. Bruder | AT-177.13 | AM502025 | AM710581 | AM710492 |
| *Psammothidium* *papilio* (D.E. Kellogg, M. Stuiver, T.B. Kellogg & G.H. Denton) K. Kopalová & B. Van de Vijver | FLB10 | KM116121.1 |  |  |
| *Psammothidium* *papilio* (D.E. Kellogg, M. Stuiver, T.B. Kellogg & G.H. Denton) K. Kopalová & B. Van de Vijver | FLB11 | KM116122.1 |  |  |
| *Reimeria* *sinuata* (Gregory) Kociolek & Stoermer | TCC719 | JN790290.1 |  |  |
| *Reimeria* *sinuata* (Gregory) Kociolek & Stoermer | TCC721 | JN790292.1 |  |  |
| *Reimeria* *sinuata* (Gregory) Kociolek & Stoermer | TCC735 | JN790291.1 |  |  |
| *Rhoicosphenia* cf. *abbreviata* (Agardh) Lange-Bertalot) | 1 EWT | KU965564 | KU965571 | KU965577 |
| *Rhoicosphenia* cf. *abbreviata* (Agardh) Lange-Bertalot) | 2 EWT | KU965565 | KU965572 | KU965578 |
| *Rhoicosphenia stoermeri* Thomas & Kociolek | 3 EWT | KU965566 | KU965573 | KU965579 |
| *Rhoicosphenia* cf. *abbreviata* (Agardh) Lange-Bertalot) | 4 EWT | KU965567 | KU965574 |  |
| *Rhoicosphenia* cf. *abbreviata* (Agardh) Lange-Bertalot) | 37 EWT | KU965568 | KU965575 |  |
| *Rhoicosphenia* cf. *abbreviata* (Agardh) Lange-Bertalot) | 80 EWT | KU965569 |  | KU965580 |
| *Rhoicosphenia* cf. *abbreviata* (Agardh) Lange-Bertalot) | 94 EWT | KU965570 | KU965576 |  |
| *Rhopalodia* *contorta* Hustedt | L1299 | HQ912406 |  | HQ912392 |
| *Rhopalodia* *gibba* (Ehrenberg) O. Müller | CH155 | HQ912407 |  | HQ912393 |
| *Rhopalodia* sp. | 9vi08.1F.2 | HQ912405 |  |  |
| *Rossia* sp. | CH2 | AJ535144 |  |  |
| *Rossia* sp. | E3333 | EF151968 |  |  |
| *Scoliopleura* *peisonis* Grunow | FD13 | HQ912609 |  |  |
| *Sellaphora* cf. *minima* (Grunow) Mann | BM42 | EF151966 |  |  |
| *Sellaphora* cf. *seminulum* (Grunow) Mann | TM37 | EF151967 |  |  |
| *Sellaphora* *pupula* (Kützing) Mereschkowsky | AUS1 | EF151982 |  | EF143312 |
| *Stauroneis* *anceps* Ehrenberg | AT-160Gel11 | AM502008 | AM710564 | AM710475 |
| *Stauroneis* *gracilior* Reichardt | AT-117Gel17 | AM501988 | AM710543 | AM710454 |
| *Stauroneis* *phoenicenteron* (Nitzsch) Ehrenberg | AT-117.04 | AM501987 | AM710542 | AM710453 |
| *Stenopterobia* *curvula* (W. Smith) Krammer | L541 | HQ912416 |  |  |
| *Surirella* *angusta* Kützing | SANG1 | AJ867028 |  |  |
| *Surirella* *minuta* Van Heurck | FD320 | HQ912658 |  | HQ912522 |
| *Surirella* *splendida* (Ehrenberg) Kützing | 19C | HQ912415 |  | HQ912401 |
| *Tryblionella* *apiculata* Gregory | FD465 | HQ912600 |  | HQ912464 |
