# Supplementary material for: Historical and Current Perspectives on the Systematics of the ‘Enigmatic’ Diatom Genus Rhoicosphenia (Bacillariophyta), with Single and Multi-Molecular Marker and Morphological Analyses and Discussion on the Monophyly of ‘Monoraphid’ Diatoms
Source: PLoS One. 2016 Apr 5;11(4):e0152797. doi: 10.1371/journal.pone.0152797 (PMC4821588; doi:10.1371/journal.pone.0152797)
Supplement: S3 Table — Characters and character states used in morphological phylogenetic analysis. (PDF) [file pone.0152797.s017.pdf]

Character #	Character	Description	Character state
1	Chloroplasts per cell	Two	0
		One	1
		Multiples of two	2
2	Chloroplast shape 1	Two-dimensional	0
		Three-dimensional	1
3	Chloroplast shape 2 (2-D shapes)	Incised plate (butterfly or simple H)	0
		Simple plate	1
		Double H-shape	2
4	Chloroplast shape 3 (3-D shapes)	Lobed with linking pyrenoid	1
		Variously lobed around a central axis	2
5	Chloroplast location	Along length of cell	0
		Fore and aft in cell	1
6	Position of centre of plastid	Under valve	0
		Against girdle	1
		Near mid-line of cell	2
7	Pyrenoid number	More than one per chloroplast	0
		One per chloroplast	1
8	Pyrenoid position in plastid	Scattered	0
		Axial	1
		Lateral	2
9	Pyrenoid shape	Curved or rounded	0
		Rod-like (angular)	1
		Tetrahedral	2
10	Valve symmetry 1	Isopolar	0
		Heteropolar	1
11	Valve symmetry 2	Bilaterally symmetrical	0
		Dorsiventral - primary side ventral	1
		Dorsiventral - primary side dorsal	2
12	Frustule symmetry	Isovalvar	0
		Heterovalvar	1
13	Valve mantle	Uniform	0
		Stepped	1
		Notched	2
14	Striae 1	Simply areolate	0
		Chambered - external surface areolate	1
		Chambered - internal surface areolate	2
15	Striae 2	Uniseriate throughout	0
		Biseriate (at least partly)	1
		Multiseriate	2
16	Areola occlusions 1	With cribra	0
		Without cribra	1
17	Areola occlusions 2	Without hymenes	0
		With hymenes	1
18	Areola occlusions 3	With volae	0
		Without volae	1
19	Areola type	Poroid	0
		Loculate	1
20	Areola openings (external) 1	More or less circular	0
		Elongate	1
		Reniform	2
21	Areola openings (external) 2	Openings discrete	0
		Openings confluent	1

Character #	Character	Description	Character state
22	Areola openings (external) 3	Opening perpendicular to stria direction	1
		Opening parallel to stria direction	2
23	Girdle bands 1	With two rows of pores	0
		With one row of pores	1
		Without pores	2
24	Girdle bands 2	Pores like valve pores	0
		Pores unlike valve pores	1
25	Internal raphe sternum	Absent	0
		With central fissure	1
		With lateral fissure	2
26	Accessory rib	Absent	0
		On primary side only	1
		On primary and secondary sides	2
27	Internal central raphe fissures 1	Unilaterally deflected	0
		Straight	1
		Oppositely deflected	2
28	Internal central raphe fissures 2	Simple	0
		Hidden (+ intermissio)	1
		Helictoglossa	2
29	Internal polar helictoglossae 1	Straight	0
		Twisted	1
		Hooded	2
30	Internal polar helictoglossae 2	Discrete	0
		Fused with sternum	1
		Forming porte-crayon ending	2
31	External central raphe endings	Straight	0
		Deflected to primary side	1
		Deflected to secondary side	2
32	External raphe endings (central v. polar)	Different	0
		Similar	1
33	External polar raphe endings	Deflected to secondary side	0
		Straight	1
		Deflected to primary side	2
		Opposite	3
34	Apical pore fields	Absent	0
		At both poles	1
		At one pole	2
35	Stigmata	None	0
		One	1
		More than one	2
