# Supplementary material for: Historical and Current Perspectives on the Systematics of the ‘Enigmatic’ Diatom Genus Rhoicosphenia (Bacillariophyta), with Single and Multi-Molecular Marker and Morphological Analyses and Discussion on the Monophyly of ‘Monoraphid’ Diatoms
Source: PLoS One. 2016 Apr 5;11(4):e0152797. doi: 10.1371/journal.pone.0152797 (PMC4821588; doi:10.1371/journal.pone.0152797)
Supplement: S4 Table — Taxa and corresponding characters used in morphological phylogenetic analysis. (PDF) [file pone.0152797.s018.pdf]

	Characters																																	
**Taxa**	**1**	**2**	**3**	**4**	**5**	**6**	**7**	**8**	**9**	**10**	**11**	**12**	**13**	**14**	**15**	**16**	**17**	**18**	**19**	**20**	**21**	**22**	**23**	**24**	**25**	**26**	**27**	**28**	**29**	**30**	**31**	**32**	**33**	**34**	**35**
Achnanthes brevipes	0	1	?	1	1	2	1	1	0	0	0	1	0	0	0	0	0	1	0	0	0	0	0	0	1	0	1	0	0	0	0	0	0	0	0
Achnanthidium minutissimum	1	0	1	0	0	1	1	1	1	0	0	1	0	0	0	1	0	1	0	0	0	0	2	1	0	0	2	0	0	0	0	1	1	0	0
Anomoeoneis sphaerophora	1	0	2	0	0	1	1	1	0	0	0	0	1	0	0	1	1	1	0	0	0	0	0	0	0	0	0	0	0	0	1	0	0	0	0
Berkeleya rutilans	1	0	0	0	0	0	1	2	0	0	0	0	0	0	0	1	1	1	0	0	0	2	0	0	0	0	1	0	0	0	2	1	0	0	0
Caloneis amphisbaena	0	0	1	0	0	1	1	1	0	0	0	0	0	1	2	1	1	1	0	0	0	0	1	1	0	0	0	1	0	0	1	0	0	0	0
Caloneis silicula	1	0	0	0	0	0	0	2	0	0	0	0	0	1	2	1	1	1	0	0	0	0	1	1	0	0	0	1	0	0	1	0	0	0	0
Climaconeis inflexa	0	1	?	1	1	2	1	1	0	0	1	0	0	0	0	1	1	1	0	0	0	0	0	0	0	1	1	0	0	0	2	1	0	0	0
Climaconeis scalaris	2	1	?	1	1	2	1	1	0	0	0	0	0	0	0	1	1	1	0	0	0	0	0	1	0	2	1	0	0	0	0	1	1	0	0
Cocconeis placentula	1	0	1	0	0	0	1	1	1	0	0	1	0	0	0	1	1	1	0	1	0	2	2	1	0	0	2	0	0	1	0	0	1	0	0
Craticula ambigua	0	0	1	0	0	1	1	1	1	0	0	0	0	0	0	1	1	1	0	1	0	1	1	1	0	0	1	0	0	0	2	0	0	0	0
Cymbella affinis	1	0	2	0	0	1	1	1	0	0	1	0	1	0	0	1	0	0	0	1	0	1	1	1	0	0	0	1	2	0	1	0	0	1	1
Cymbella cymbiformis	1	0	2	0	0	1	1	1	0	0	1	0	1	0	0	1	0	0	0	1	0	1	1	1	0	0	0	1	2	0	1	0	0	1	2
Cymbella lanceolata	1	1	?	2	0	2	1	1	0	0	1	0	1	0	0	1	0	0	0	1	0	1	1	1	0	0	0	1	2	0	1	0	0	1	2
Encyonema caespitosum	1	0	2	0	0	1	1	1	0	0	2	0	1	0	0	1	0	1	0	1	0	1	1	1	0	0	0	0	0	0	1	0	0	0	0
Encyonema prostratum	1	0	2	0	0	1	1	1	0	0	2	0	1	0	0	1	0	1	0	1	0	1	1	1	0	0	0	0	0	0	1	0	0	0	0
Gomphonema acuminatum	1	0	2	0	0	1	1	1	0	1	0	0	1	0	1	1	0	0	0	2	0	1	1	1	0	0	0	0	0	0	1	0	0	2	1
Gomphonema parvulum	1	0	2	0	0	1	1	1	0	1	0	0	1	0	0	1	0	0	0	2	0	1	1	1	0	0	0	0	0	0	0	0	0	2	1
Mastogloia smithii	0	1	?	1	1	2	1	1	0	0	0	0	0	0	0	0	0	1	0	0	0	0	0	0	1	0	1	0	0	1	2	0	0	0	0
Navicula gregaria	0	0	1	0	0	1	1	1	1	0	0	0	0	0	0	1	1	1	1	1	0	1	2	1	2	1	1	0	1	1	1	0	0	0	0
Navicula tripunctata	0	0	1	0	0	1	1	1	1	0	0	0	0	0	0	1	1	1	1	1	0	1	2	1	2	1	1	0	1	1	0	0	0	0	0
Pinnularia gibba	0	0	1	0	0	1	1	1	0	0	0	0	0	1	2	1	1	1	0	0	0	0	1	1	0	0	0	1	0	0	1	0	0	0	0
Pinnularia lundii	1	0	0	0	0	0	0	2	0	0	0	0	0	1	2	1	1	1	0	0	0	0	1	1	0	0	0	0	0	0	0	0	0	0	0
Pinnularia viridis	0	0	1	0	0	1	?	?	?	0	0	0	0	1	2	1	1	1	0	0	0	0	1	1	0	0	0	1	0	0	1	0	0	0	0
Placoneis clementioides	1	1	?	2	0	2	1	1	0	0	0	0	1	0	0	1	0	0	0	0	0	0	1	0	0	0	0	0	0	0	0	0	3	0	2
Placoneis gastrum	1	1	?	2	0	2	1	1	0	0	0	0	1	0	0	1	0	0	0	0	0	0	1	0	0	0	0	0	0	0	0	0	0	0	0
Placoneis placentula	1	1	?	2	0	2	1	1	0	0	0	0	1	0	1	1	0	0	0	0	0	0	1	0	0	0	0	0	0	0	0	0	3	0	2
Reimeria sinuata	1	0	2	0	0	1	1	1	0	0	2	0	1	0	1	1	0	0	0	0	0	0	1	1	0	0	0	0	0	0	0	0	0	1	1
Rhoicosphenia curvata	1	0	2	0	0	1	1	1	0	1	0	1	0	0	0	1	1	1	0	1	0	1	1	0	0	0	0	0	0	0	0	0	0	2	0
Sellaphora bacillum	1	0	0	0	0	0	1	2	2	0	0	0	0	0	0	1	1	1	0	0	0	0	2	1	0	0	0	0	0	0	1	0	0	0	0
Sellaphora pupula	1	0	0	0	0	0	1	2	2	0	0	0	0	0	0	1	1	1	0	0	0	0	2	1	0	0	0	0	0	0	1	0	0	0	0
Stauroneis anceps	0	0	1	0	0	1	0	1	0	0	0	0	0	0	0	1	1	1	1	1	0	2	1	1	0	0	1	0	0	0	2	0	0	0	0
Stauroneis phoenicenteron	0	0	1	0	0	1	0	1	0	0	0	0	0	0	0	1	1	1	1	1	0	2	1	1	0	0	1	0	0	0	2	0	0	0	0
Stauroneis smithii	0	0	1	0	0	1	1	1	0	0	0	0	0	0	0	1	1	1	1	1	0	1	1	1	0	0	1	0	?	?	0	0	0	0	0
